# Machine learning insights into thrombo-ischemic risks and bleeding events through platelet lysophospholipids and acylcarnitine species

**DOI:** 10.1038/s41598-024-56304-x

**Published:** 2024-03-13

**Authors:** Tobias Harm, Xiaoqing Fu, Moritz Frey, Kristina Dittrich, Adrian Brun, Tatsiana Castor, Oliver Borst, Karin Anne Lydia Müller, Tobias Geisler, Dominik Rath, Michael Lämmerhofer, Meinrad Paul Gawaz

**Affiliations:** 1 Department of Cardiology and Angiology, University Hospital Tübingen, Eberhard Karls University Tübingen, Otfried-Müller-Straße 10, 72076 Tübingen, Germany; 2 Institute of Pharmaceutical Sciences, Eberhard Karls University Tübingen, Auf der Morgenstelle 8, 72076 Tübingen, Germany

**Keywords:** Platelets, Acute coronary syndromes, Machine learning, Metabolomics, Predictive markers

## Abstract

Coronary artery disease (CAD) often leads to adverse events resulting in significant disease burdens. Underlying risk factors often remain inapparent prior to disease incidence and the cardiovascular (CV) risk is not exclusively explained by traditional risk factors. Platelets inherently promote atheroprogression and enhanced platelet functions and distinct platelet lipid species are associated with disease severity in patients with CAD. Lipidomics data were acquired using mass spectrometry and processed alongside clinical data applying machine learning to model estimates of an increased CV risk in a consecutive CAD cohort (*n* = 595). By training machine learning models on CV risk measurements, stratification of CAD patients resulted in a phenotyping of risk groups. We found that distinct platelet lipids are associated with an increased CV or bleeding risk and independently predict adverse events. Notably, the addition of platelet lipids to conventional risk factors resulted in an increased diagnostic accuracy of patients with adverse CV events. Thus, patients with aberrant platelet lipid signatures and platelet functions are at elevated risk to develop adverse CV events. Machine learning combining platelet lipidome data and common clinical parameters demonstrated an increased diagnostic value in patients with CAD and might improve early risk discrimination and classification for CV events.

## Introduction

Platelet hyperreactivity is an important risk factor for development of acute coronary thrombosis and adverse thrombo-ischemic events in patients suffering from coronary artery disease (CAD)^1–6^. Further, platelet hyperresponsiveness contributes to atheroprogression in patients with CAD^1,7,8^. Beyond atherogenic mechanisms involved in progression of CAD, sustained platelet activation and thrombo-inflammation are associated with adverse thrombo-ischemic events in patients with both chronic coronary syndrome (CCS) and acute coronary syndrome (ACS)^2^.

In patients with diabetes mellitus and dyslipidemia enhanced levels of blood glucose and lipoproteins, such as low-density lipoprotein (LDL), trigger thrombo-inflammatory signaling cascades leading to platelet activation^9–11^. Further, platelet lipid cargo including uptake of oxidized LDL promotes platelet hyperreactivity and impacts the platelet lipid signature^9^.

Recent advances in mass spectrometry enabled to characterize changes of the platelet lipidome and to identify key lipids essential for platelet function^12–18^. Recently, significant changes in the platelet lipidome were observed between patients with ACS and CCS^9,19^. In patients with adverse CV events distinct platelet lipid species accelerate disease severity in patients with ACS^19,20^. Only recently, we depicted that platelet levels of lysophosphatidylethanolamine (LPE) and acylcarnitines (CAR) were independently associated with adverse thrombo-ischemic and bleeding events in patients with cardiovascular disease (CVD)^20,21^.

The composition of the platelet lipidome correlated with lipid-lowering treatment and lipid subspecies including LPE are susceptible to statin treatment^21,22^. Thus, the platelet lipidome signature may offer the perspective to identify patients at risk and to attenuate the burden of adverse clinical events in CAD. In this study we performed machine learning models employing platelet lipidomics profiling with mass spectrometry coupled to liquid-chromatography in a large (*n* = 595) cohort study of patients with symptomatic CAD. We stratify the cardiovascular risk by analyzing distinct lipid species and platelet function over three years in the prospective population-based study.

## Results

### Determination of sub-phenotypes in patients with coronary artery disease at elevated cardiovascular risk

**Figure 1:**
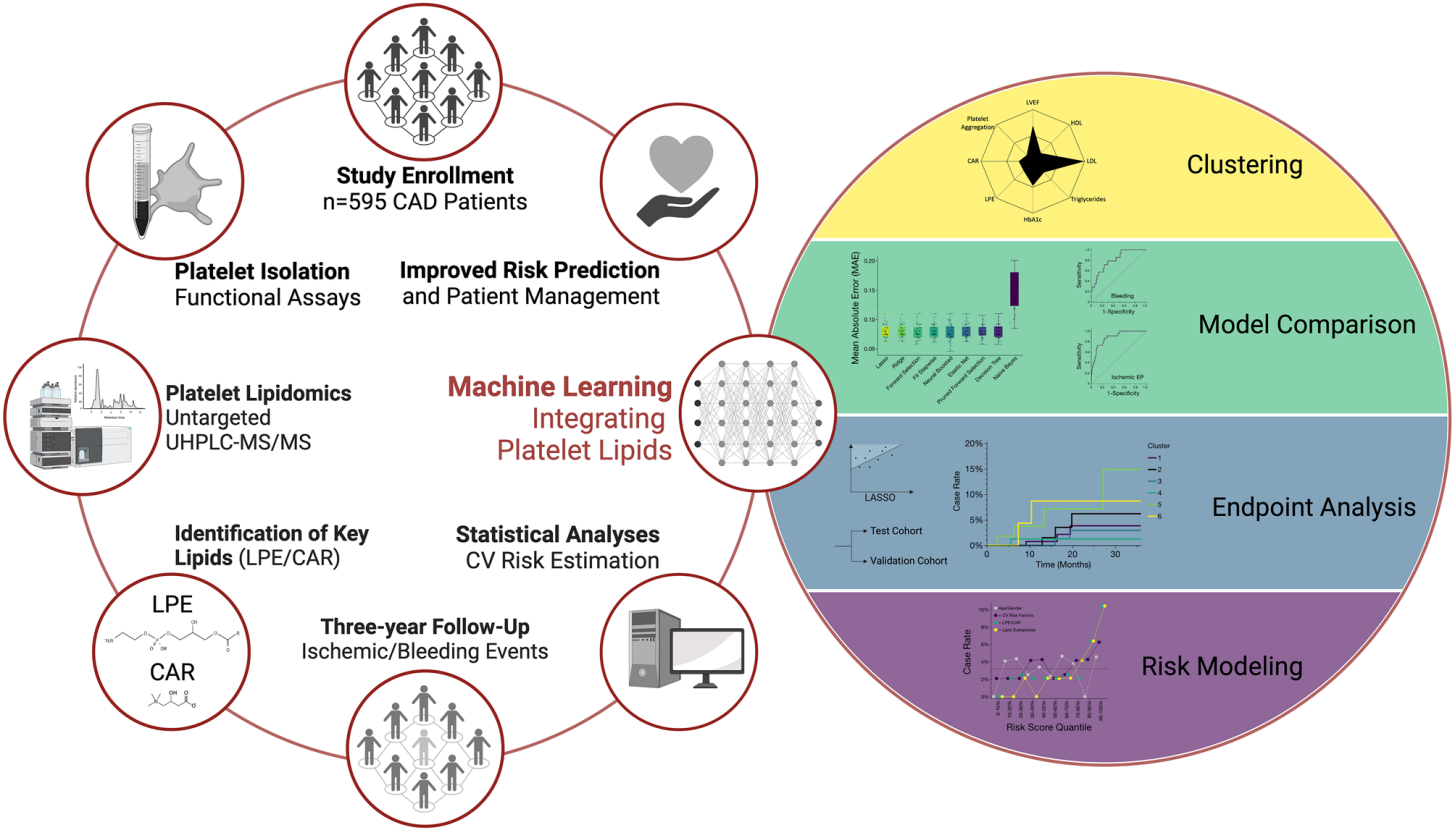
Machine learning of cardiovascular risk factors including the platelet lipidome facilitates sub-phenotyping and prediction of adverse events in patients with CAD. Workflow of this large-scale (*n* = 595) prospective study investigating the significance of the platelet lipidome to predict adverse thrombo-ischemic and bleeding events in patients with CAD by machine learning. The platelet lipidome in this study was assessed though an untargeted UHPLC-MS/MS assay. Alongside reliable risk parameters including platelet functional data, platelet lipids significantly contributed to risk prediction of adverse thrombo-ischemic and major bleeding events during the three-year clinical follow-up. CAR, acylcarnitines; LPE, lysophosphatidylethanolamines; UHPLC-MS/MS, Ultra-high performance liquid chromatography tandem mass spectrometry.

In the present study we prospectively analyzed cardiovascular risk factors including platelet lipid signatures in a large-scale cohort of patients with CAD utilizing an untargeted UHPLC-ESI-QTOF-MS/MS approach (Fig. 1). Patients baseline characteristics including clinical and laboratory parameters of the cohort (*n* = 595) are summarized in Table 1. Over a median follow-up period of three years, 41 individuals (6.9%) experienced a major thrombo-ischemic or major bleeding event (Table 2). Recently, we described a significant association of platelet lysophosphatidylethanolamines (LPE) and acylcarnitines (CAR) upregulation with adverse ischemic or bleeding events in patients with CAD^21^.

**Table 1:** Baseline characteristics of CAD patient population. Significant values (*p* < 0.05) are highlighted.

	All	No adverse events	Adverse events	p-value
	(*n* = 595)	(*n* = 554; 93.1%)	(*n* = 41; 6.9%)	
Female, n (%)	174 (29.2)	166 (30)	8 (19.5)	0.156
Age, years (mean ± SD)	70 (± 11.4)	69.7 (± 11.4)	74 (± 11.4)	**0.026**
Body mass index (mean ± SD)	27.4 (± 5.1)	27.5 (± 5.1)	26.8 (± 4.3)	0.308
Cardiovascular risk factors				
Arterial hypertension, *n* (%)	532 (89.4)	496 (89.5)	36 (87.8)	0.729
Hyperlipidemia, *n* (%)	473 (79.5)	440 (79.4)	33 80.5)	0.871
Diabetes mellitus, *n* (%)	193 (32.4)	178 (32.1)	15 (36.6)	0.557
HbA1c (%) (mean ± SD)	6.3 (± 1.1)	6.3 (± 1.1)	6.4 (± 1.2)	0.736
Current smoking, *n* (%)	113 (19.1)	108 (19.6)	5 (12.2)	0.246
Ex Smoking > 6 mo, *n* (%)	129 (21.8)	119 (21.6)	10 (24.4)	0.672
Obesity, *n* (%)	139 (23.4)	129 (23.3)	10 (24.4)	0.671
Atrial Fibrillation, *n* (%)	139 (23.4)	126 (22.8)	13 (31.7)	0.195
Previous CABG, *n* (%)	22 (3.7)	19 (3.4)	3 (7.3)	0.203
Previous MI, *n* (%)	131 (22)	123 (22.2)	8 (19.5)	0.688
Renal function (GFR) (mean ± SD)	80.2 (± 28.8)	80.4 (± 28.8)	78.1 (± 29.8)	0.638
Medication on admission				
Statins, *n* (%)	497 (83.5)	462 (83.4)	35 (85.4)	0.743
Ezetimibe, *n* (%)	83 (13.9)	79 (14.3)	4 (9.8)	0.420
Acetylsalicylic acid, *n* (%)	543 (91.3)	508 (91.7)	35 (85.4)	0.166
Clopidogrel, *n* (%)	264 (44.4)	249 (45)	15 (36.6)	0.299
Ticagrelor, *n* (%)	133 (22.4)	124 (22.4)	9 (22)	0.944
Prasugrel, *n* (%)	112 (18.9)	107 (19.4)	5 (12.2)	0.259
Cangrelor, *n* (%)	1 (0.2)	1 (0.2)	0 (0)	0.785
Oral anticoagulants, *n* (%)	130 (21.9)	116 (20.9)	14 (34.2)	0.060
Angiotensin-converting enzyme inhibitors, *n* (%)	194 (32.6)	182 (32.9)	12 (29.3)	0.637
Angiotensin II receptor antagonists, *n* (%)	312 (52.4)	292 (52.7)	20 (48.8)	0.627
Aldosterone antagonists, *n* (%)	152 (25.6)	142 (25.6)	10 (24.4)	0.860
Ca channel antagonists, *n* (%)	211 (35.5)	196 (35.4)	15 (36.6)	0.876
β-blockers, *n* (%)	418 (70.3)	386 (69.9)	32 (78.1)	0.258
Diuretics, *n* (%)	209 (35.2)	194 (35.1)	15 (36.6)	0.846
Lipid profile parameters				
LDL-cholesterol (mg/dL) (mean ± SD)	107.5 (± 43.8)	107.5 (± 43.5)	107.4 (± 47.2)	0.988
HDL-cholesterol (mg/dL) (mean ± SD)	45.5 (± 16.2)	45.4 (± 16.3)	46.5 (± 16.2)	0.689
Triglycerides (mg/dL) (mean ± SD)	141.1 (± 87.2)	142.6 (± 88.3)	120.3 (± 68.7)	0.055
Total cholesterol (mg/mL) (mean ± SD)	165.8 (± .45.4)	165.8 (± 44.8)	164. (± 52.7)	0.908
Platelets (mean ± SD)	227.6 (± 65.9)	228.5 (± 65.6)	215.9 (± 68.4)	0.261
Disease				
Chronic coronary syndrome, *n* (%)	227 (38.2)	210 (37.9)	17 (41.5)	0.651
Unstable Angina, *n* (%)	87 (14.6)	85 (15.3)	2 (4.9)	0.067
NSTEMI, *n* (%)	210 (35.3)	191 (34.5)	19 (46.3)	0.125
STEMI, *n* (%)	71 (11.9)	68 (12.3)	3 (7.3)	0.345

**Table 2:** Clinical endpoints at three-year follow-up.

36 Months follow-up	Ischemic endpoint (*n* = 25)	Bleeding endpoint (*n* = 16)
Cardiac death, *n* (%)	7 (28)	
Myocardial infarction, *n* (%)	8 (32)	
Stroke, *n* (%)	10 (40)	
Major bleeding, *n* (%)		16 (100)

**Figure 2:**
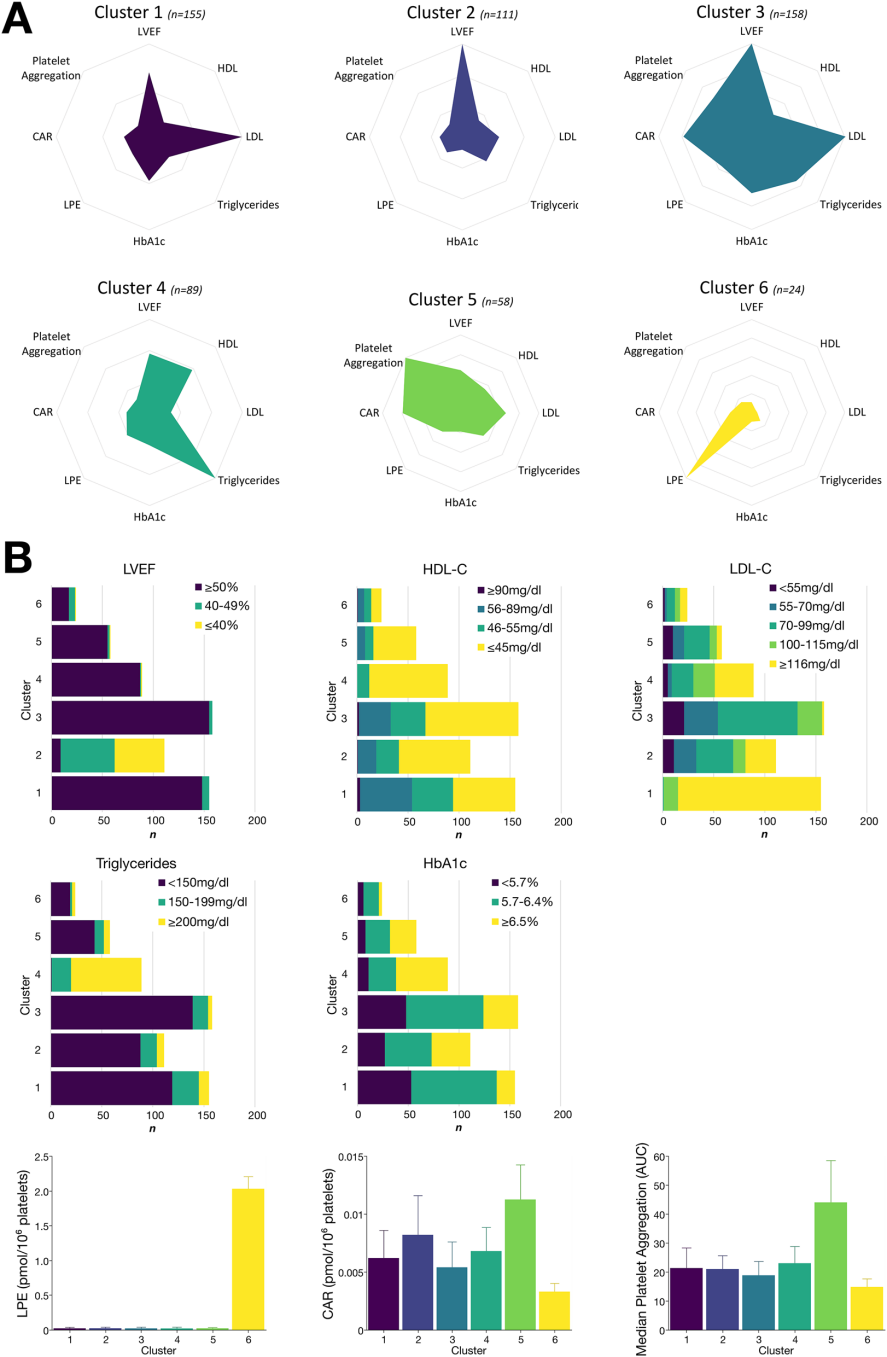
Machine learning of cardiovascular risk factors including the platelet lipidome facilitates sub-phenotyping of CAD patients. **(A)** Medoid clustering with the corresponding standardized level (z scores) of the feature risk variables (LVEF, left ventricular ejection fraction; HDL, high-density lipoprotein; LDL, low-density lipoprotein; triglycerides; HbA1c; LPE, lysophosphatidylethanolamines; CAR, acylcarnitines; platelet aggregation). Remarkably, patients summarized in cluster 5 mainly showed aberrant platelet function and enhanced platelet CAR concentrations, whereas cluster 6 was exclusively characterized by increased LPE concentrations. **(B)** Number of patients with CAD by cluster according to conventional risk parameter with color indicating cut-off values of individual measurements. In addition, alongside median platelet LPE and CAR concentrations, median area under the curve (AUC) from merged collagen-, arachidonic acid-, adenosine diphosphate-, and thrombin-induced platelet aggregation was depicted by cluster to identify patients with platelet hyperreactivity and aberrant platelet lipid signatures. Error bars were constructed based on interquartile range (IQR).

Initial clustering and identification of sub-phenotypes in CAD patients was done integrating CV risk factors (LDL and HDL cholesterol, triglycerides, HbA1c, LVEF, and platelet aggregation) as well as mean platelet LPE and CAR concentrations. We could identify six clusters with distinctive patterns of the variables (Fig. 2). Cluster characteristics regarding CV risk parameters are depicted in Fig. 2 and Table 3. Patients grouped into cluster 5 shared a relative high abundance of platelet CAR levels as well as platelet hyperreactivity, cluster 6 was solely characterized by critically enhanced LPE concentrations (Fig. 2). Further, the number of patients with impaired LVEF was highest in cluster 2, LDL cholesterol was highest in cluster 1, whereas patients in cluster 4 showed high levels of HbA1c, elevated triglycerides and low HDL cholesterol (Fig. 2). Of note, in patient clusters 1–5, lipids sharing the highest abundance were lysophosphatidylethanolamines LPE18:0/0:0, LPE P-18:0, and LPE 0:0/20:4. The latter exhibited highest concentration in patient cluster 6 and was followed by LPE 0:0/22:4 and LPE 0:0/22:5. Overall, characteristic LPE with side chain length of 18 carbon atoms showed highest concentrations among all patients in this study (Supplementary Figure S2). Important cluster characteristics and subgroup comparisons are shown in Table 3, and in-depth juxtaposition of each cluster is outlined in supplementary Table 2 and supplementary Figures S5 & S6.

**Table 3:** Cluster characteristics of the CAD patient cohort.

	Cluster 1 (*n* = 155)	Cluster 2 (*n* = 111)	Cluster 3 (*n* = 158)	Cluster 4 (*n* = 89)	Cluster 5 (*n* = 58)	Cluster 6 (*n* = 24)	p-Value
Female, *n* (%)	55 (35.5)	28 (25.2)	40 (25.2)	26 (29.2)	15 (25.9)	10 (41.7)	0.215
Age, years (mean ± SD)	69 (± 12.3)	71.8 (± 12.3)	71.4 (± 10.3)	64.7 (± 11.2)	72.7 (± 10.5)	73.4 (± 11)	6.9*10^**−6**^
Body mass index (mean ± SD)	26.4 (± 5.4)	27 (± 5.1)	27.3 (± 4.7)	30.3 (± 5)	27.7 (± 4.4)	25.9 (± 3.3)	6.3*10^**−7**^
Disease							
Chronic coronary syndrome, *n* (%)	48 (31)	34 (30.6)	80 (50.6)	25 (28.1)	30 (51.7)	10 (41.7)	**0.001**
Unstable angina, *n* (%)	22 (14.2)	10 (9)	25 (15.8)	22 (24.7)	8 (13.8)	0 (0)	**0.013**
NSTEMI, *n* (%)	64 (41.3)	50 (45.1)	44 (27.9)	27 (30.3)	16 (27.6)	9 (37.5)	**0.020**
STEMI, *n* (%)	21 (13.6)	17 (15.3)	9 (5.7)	15 (16.9)	4 (6.9)	5 (20.8)	**0.026**
Cardiovascular risk factors							
Arterial hypertension, *n* (%)	133 (85.8)	98 (88.3)	145 (91.2)	84 (94.4)	51 (87.5)	21 (89.4)	0.331
Hyperlipidemia, *n* (%)	132 (85.2)	86 (77.5)	117 (74)	75 (84.3)	42 (72.4)	21 (87.5)	0.070
Diabetes mellitus, *n* (%)	22 (14.2)	45 (40.54)	43 (27.2)	51 (57.3)	28 (48.3)	4 (16.67)	2.5*10^**−12**^
Current smoking, *n* (%)	31 (20.1)	21 (18.9)	24 (15.3)	26 (29.2)	6 (10.3)	5 (20.8)	0.064
Ex Smoking > 6 mo, *n* (%)	32 (20.8)	31 (27.9)	28 (17.8)	19 (21.4)	16 (27.6)	3 (12.5)	0.272
Obesity, *n* (%)	24 (15.5)	19 (17.1)	35 (22.2)	46 (51.7)	13 (22.4)	2 (8.3)	9.3*10^**−6**^
Atrial fibrillation, *n* (%)	28 (18.2)	34 (30.6)	31 (19.8)	12 (13.5)	26 (44.8)	8 (33.3)	4.0*10^**−5**^
Previous CABG, *n* (%)	1 (0.6)	7 (6.3)	5 (3.2)	5 (5.6)	3 (5.2)	1 (4.2)	0.180
Previous MI, *n* (%)	13 (8.4)	44 (39.6)	34 (21.5)	25 (28.1)	12 (20.7)	3 (12.5)	1.4*10^**−7**^
Laboratory parameters							
LDL-cholesterol (mg/dL) (mean ± SD)	153.5 (± 31.3)	96.1 (± 39.7)	77 (± 19.1)	115 (± 38.2)	78.8 (± 26.4)	105.3	2.5*10^**−80**^
HDL-cholesterol (mg/dL) (mean ± SD)	53.1 (± 17.8)	44.3 (± 16)	45.4 (± 15.2)	34.8 (± 7.4)	40.9 (± 12.8)	53.1 (± 17.6)	9.9*10^**−18**^
Triglycerides (mg/dL) (mean ± SD)	124.1 (± 52.4)	115.2 (± 51.6)	108 (± 38.7)	278.6 (± 121)	123.5 (± 53.8)	120.6 (± 69.5)	1.44*10^**−70**^
Total cholesterol (mg/mL) (mean ± SD)	210.1 (± 34.1)	151.5 (± 41.8)	136 (± 23.6)	178.6 (± 37.8)	134.3 (± 31.5)	170.1 (± 44.4)	6.2*10^**−72**^
HbA1c (%) (mean ± SD)	5.9 (± 0.7)	6.5 (± 1.3)	6.1 (± 0.8)	7 (± 1.5)	6.5 (± 1.1)	6 (± 0.5)	4.1*10^**−15**^
Renal function (GFR) (mean ± SD)	83.3 (± 22.1)	72.6 (± 27.8)	80.5 (± 35.9)	82.5 (± 26.8)	80.9 (± 29.6)	83.5 (± 18.9)	0.0663
LVEF (%) (mean ± SD)	57 (± 5.2)	37 (± 6.3)	58.2 (± 4)	57.2 (± 5.4)	56.7 (± 6)	52.8 (± 8.7)	3.6*10^**−145**^
Platelets (10^9^/l) (mean ± SD)	235.4 (± 53.3)	230.6 (± 76.8)	211.2 (± 64.3)	235.3 (± 68.4)	226.7 (± 64.2)	245.4 (± 74.4)	**0.010**
Platelet Aggregation (AUC) (mean ± SD)	22.5 (± 10.6)	21.2 (± 11)	19 (± 6.8)	23.3 (± 9.6)	45.3 (± 18.3)	16.4 (± 8.7)	6.3*10^**−49**^
Platelet lipidomics							
LPE (pmol/10^9^ platelets) (mean ± SD)	43.5 (± 54)	41.1 (± 43.9)	41.3 (± 61.1)	37.5 (± 42)	38.3 (± 56.2)	1988.1 (± 303.3)	9.8*10^**−11**^
CAR (pmol/10^9^ platelets) (mean ± SD)	6.7 (± 2.9)	8.7 (± 4.1)	5.8 (± 2.5)	7 (± 3)	11 (± 5.6)	3.7 (± 1.5)	7.2*10^**−27**^

Significant values are in [bold].

**Figure 3:**
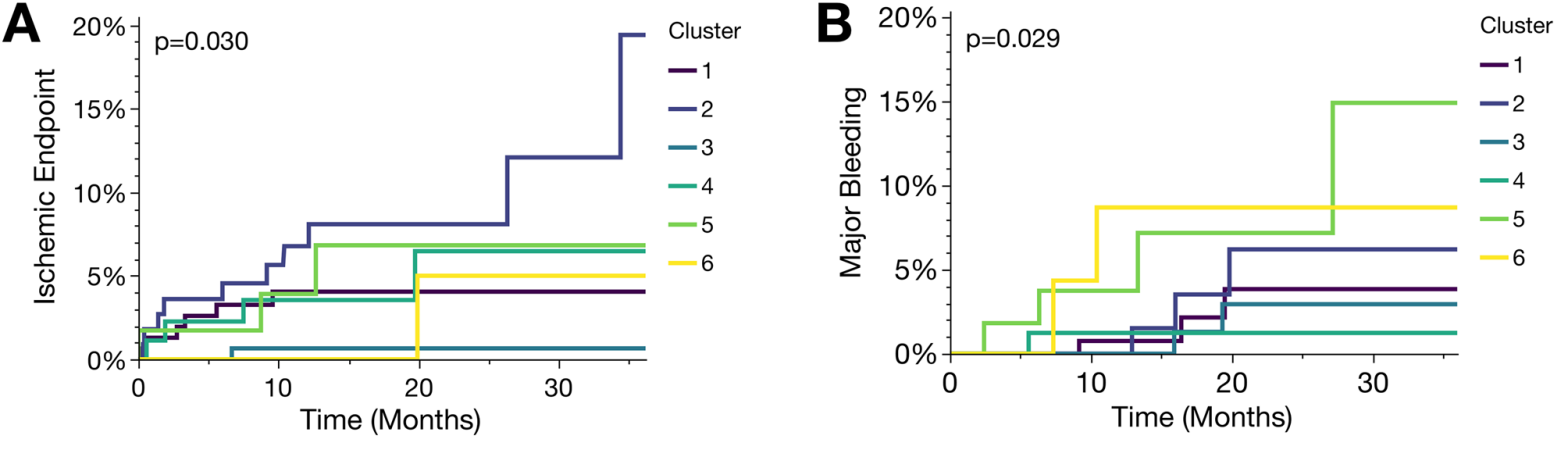
Patients with coronary artery disease and aberrant platelet lipid signatures are at increased risk to develop adverse cardiovascular events. Kaplan–Meier curves showing cluster-specific probability to develop adverse ischemic (**A**; ischemic stroke, myocardial infarction, CV death) or major bleeding events (**B**), respectively. Failure curves were significantly (*p* < 0.05) divergent between cluster groups. *N* = 595, mean follow-up 36 months, number of adverse ischemic events *n* = 25 and number of bleeding events *n* = 16.

In the longitudinal analysis, all participants were screened for major bleeding or ischemic events (Fig. 3). We found that bleeding incidence was the highest in cluster 5, followed by cluster 6, whereas the incidence of ischemic events was highest in cluster 2, followed by cluster 5. Here, it was noticeable that patients in cluster 6 with exclusively elevated platelet LPE concentrations showed an increased incidence for both, ischemic and bleeding events (Fig. 3). The composite endpoint including both, bleeding and ischemic events revealed highest incidences in cluster 2 followed by cluster 5 and 6 (Supplementary Figure S3). Overall-mortality during the three-year follow-up was highest in cluster 2 and 5 (Supplementary Figure S3). Of note, adverse events were not enriched in patients with ACS (e.g. STEMI, NSTEMI, unstable angina) when compared to patients with CCS (Supplementary Figure S4).

To test which parameters independently predict an elevated incidence of both, ischemic and bleeding events, respectively, we performed Cox proportional hazard analyses (Table 4). On the one hand, CAR concentration (HR 21.89, 95% CI 1.38–346.4, *p* = 0.029) and LPE/CAR ratio (HR 90.83, 95% CI 2.2–3745.32, *p* = 0.018) were independent predictors of an increased CV risk for adverse ischemic events (Table 4). On the other hand, mean platelet CAR concentration (HR 162.35, 95% CI 6.91–3816.13, *p* = 0.002) was independently associated with incident bleeding (Table 4). Same results were replicable in a simplified Cox proportional hazard model using only mean platelet LPE and CAR concentrations as well as the corresponding LPE/CAR ratio (Supplementary Tables S3 and S4).

**Table 4:** Improved assessment of thrombo-ischemic and major bleeding events in patients with CAD integrating platelet lipid species.

Variables	Ischemic Endpoint (*n* = 25)		Bleeding Endpoint (*n* = 16)	
	HR (95% CI)	*p*-Value	HR (95% CI)	*p*-Value
LVEF	0.37 (0.12–1.12)	0.079	2.25 (0.36–14.3)	0.378
HbA1c	5.69 (0.3–107.89)	0.247	0.03 (0.01–15.81)	0.261
Platelet aggregation	2.1 (0.21–21.17)	0.530	4.-6 (0.28–87.6)	0.274
LDL	4.67 (0.67–32.63)	0.120	0.78 (0.05–12.95)	0.865
HDL	4.47 (0.21–94.1)	0.335	0.16 (0.01–21.85)	0.462
Triglycerides	0.43 (0.01–65.25)	0.743	0.01 (9.3e^**−**10^–19.98)	0.143
LPE	0.3 (0.01–4.3)	0.377	3.07 (0.33–29.58)	0.332
CAR	21.89 (1.38–346.4)	**0.029**	162.35 (6.91–3816.13)	**0.002**
LPE/CAR Ratio	90.83 (2.2–3745.32)	**0.018**	5.93 (0.13–265.18)	0.359

Cox proportional hazard model including platelet lipid signatures. Variables significantly (*p* < 0.05) contributing to the prediction of adverse events during the three-year follow-up are highlighted. HR, hazard ratio; CI, confidence interval; LVEF, left ventricular ejection fraction; LDL, low-density lipoprotein; HDL, high-density lipoprotein; LPE, lysophosphatidylethanolamines; CAR, acylcarnitines.

### Machine learning of important risk factors including platelet lipid signatures to predict an increased cardiovascular risk

**Figure 4:**
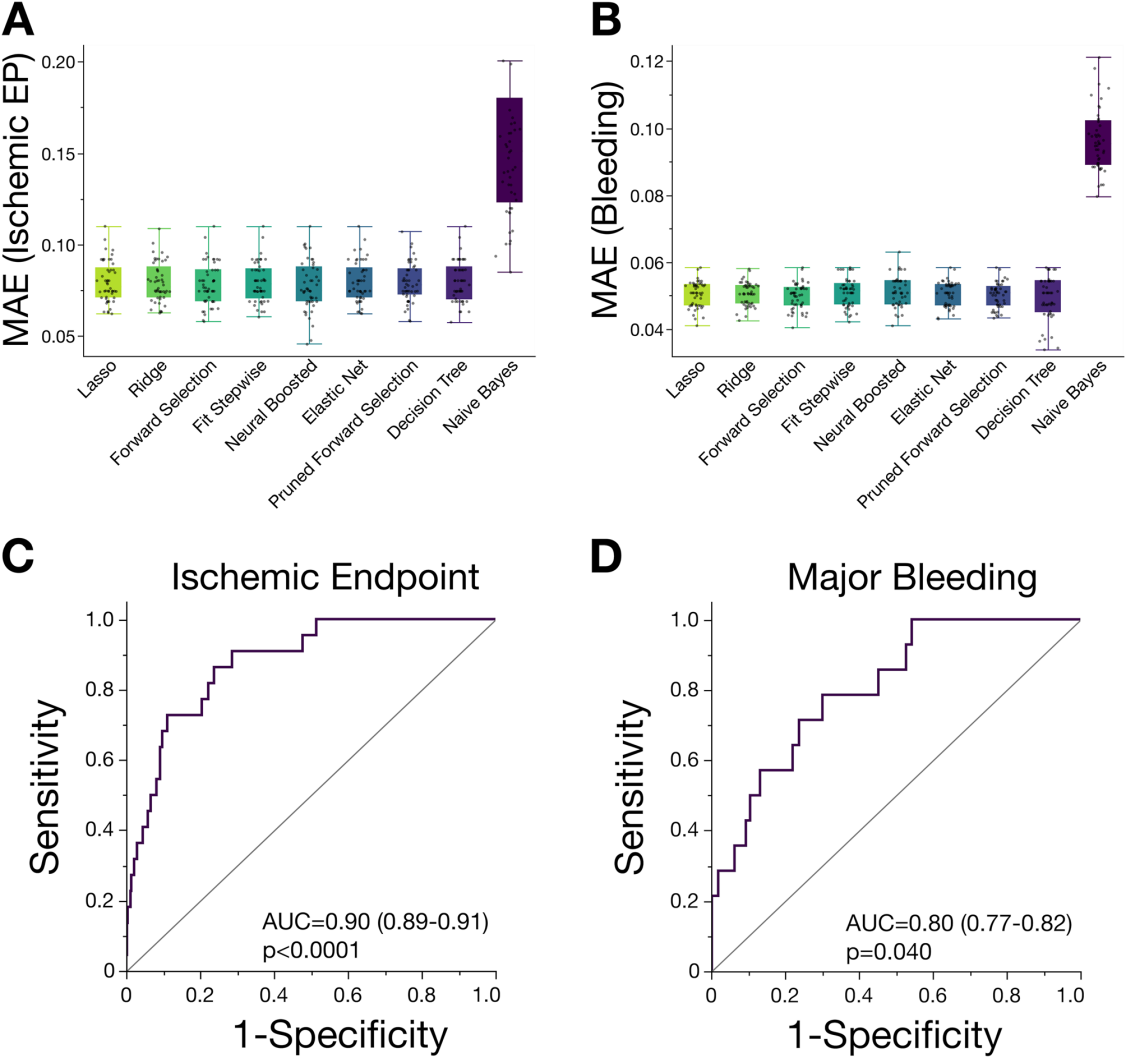
Machine learning of cardiovascular risk factors including the platelet lipidome in patients with CAD enhances the diagnostic accuracy of CV risk prediction. Comparison machine-learning algorithms on platelet lipidomics data showing mean absolute error (MAE) of predicting adverse ischemic (**A**) and major bleeding (**B)** events in patients with CAD. Least absolute shrinkage and selection operator (LASSO) showed a superior MAE among regression models and was implemented for further analyses. (**C)** Receiver operator characteristic (ROC) plot of the final LASSO model including platelet lipid subspecies (lysophosphatidylethanolamines (LPE) and acylcarnitines (CAR)) to predict adverse ischemic events. (**D)** ROC plot of the final LASSO model including platelet LPE/CAR to predict major bleeding events.

To derive new lipid species in patients with CAD, being eligible to sufficiently determine the risk of both, adverse bleeding, or thrombo-ischemic events, we compared mean absolute errors (MAE) using different regression algorithms on molar lipid concentration data in this study. The least absolute shrinkage and selection operator (LASSO) model showed a robust goodness of fit to compute the data and was therefore implemented for further analyses (Fig. 4A,B). Thus, overfitting of potential risk factors and lipids is minimized and thus, variable selection and regularization is optimized leading to an enhanced prediction accuracy. Therefore, cross-validated LASSO selection algorithm was performed including cardiovascular risk factors (i.e. LDL-cholesterol, HDL-cholesterol, triglycerides, LVEF, HbA1c and platelet function). In the L_1_-regularised model assessing the risk of adverse ischemic events, nine lipids shared nonzero coefficients and thus were related to incident CVD. CAR 5:0, CAR 10:0, CAR 14:0, CAR 16:0, LPE 0:0/18:0, LPE 18:0/0:0 and LPE 20:1/0:0 were included into the model to predict thrombo-ischemic events. LASSO model showed a high prediction accuracy (AUC = 0.9, 95% CI 0.89–0.91, *p* < 0.0001) (Fig. 4C). When assessing the risk of bleeding events in patients with CAD, LASSO algorithm included fourteen lipids with a nonzero coefficient. Likewise, the model comprising of CAR 5:0, CAR 8:0, CAR 10:0, CAR 14:0, CAR 14:1, CAR 16:0, CAR 16:1, LPE 16:0/0:0, LPE 20:0, LPE 20:4, LPE 20:5 and LPE P-16:0 indicated a high accuracy to predict bleeding evens (AUC = 0.8, 95% CI 0.77–0.82, *p* = 0.04) (Fig. 4D).

### Platelet lipid species are significantly related and predict clinical characteristics in patients with coronary artery disease

**Figure 5:**
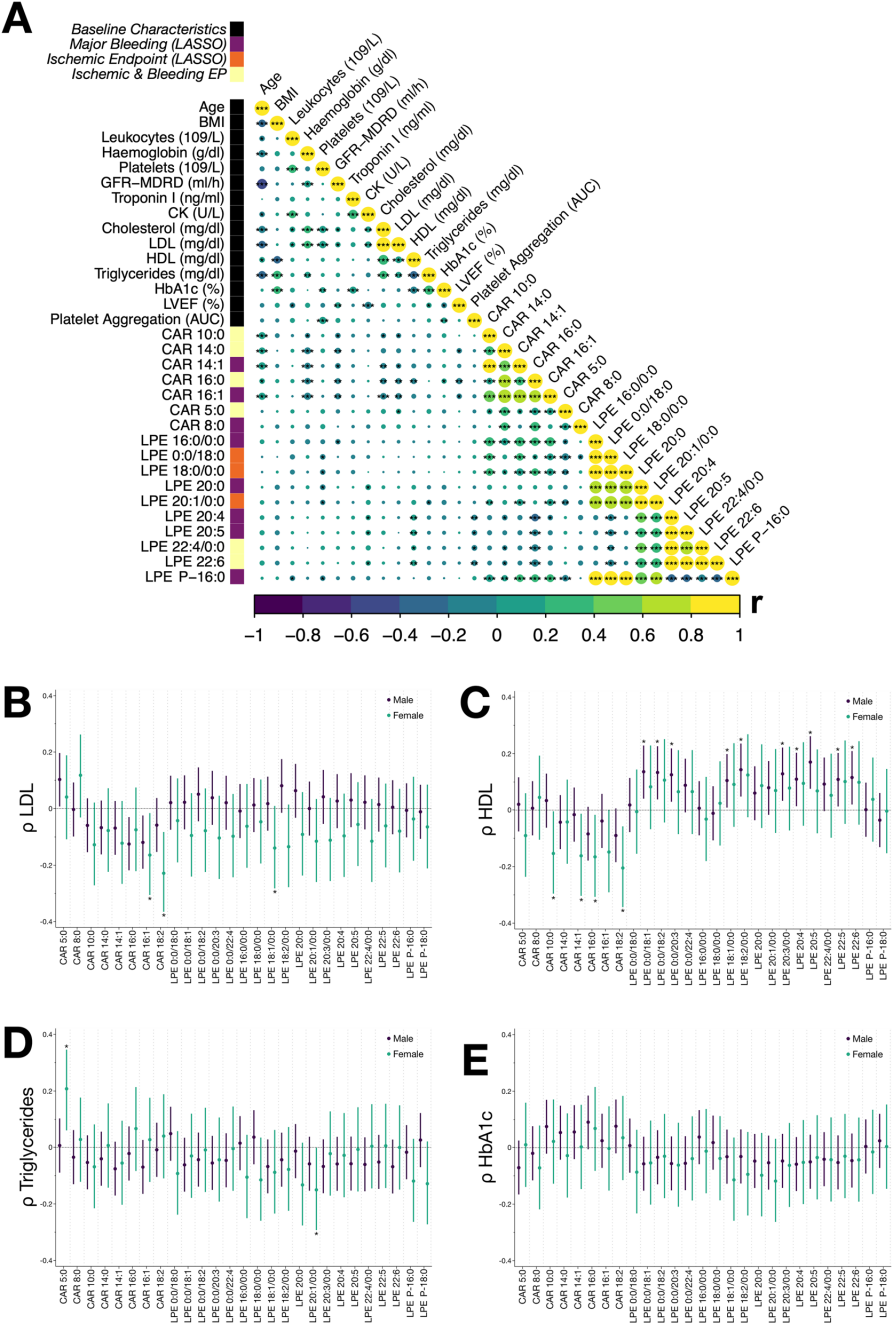
Platelet lipid species correlate with clinical parameters in patients with coronary artery disease. (**A)** Comprehensive correlation matrix of clinical parameters alongside platelet lipidomics data. Spearman's ρ is color accordingly and significant values (*p < 0.05, **p < 0.01, ***p < 0.001) are labeled. Platelet lipids belonging to the class of lysophosphatidylethanolamines (LPE) or acylcarnitines (CAR) integrated into LASSO models are colored accordingly. (**B-E)** Correlation analyses of platelet LPE and CAR with concentrations of LDL, HDL, triglycerides and HbA1c. Pearson correlation coefficients (*ρ*) and their 95% CI for each sex are shown for lipid subspecies. Significant correlations (*p* < 0.05) are highlighted.

As described recently, the platelet lipidome of patients with adverse CV events is characterized by altered LPE and CAR concentrations, when compared to patients without incident CVD^21^. Thus, we hypothesized that changes of the platelet lipidome correlate with important clinical parameters and, therefore, we performed comprehensive correlation analysis of important clinical baseline characteristics and platelet lipid species included in the LASSO prediction model. We found that individual platelet LPE and CAR lipids significantly (*p* < 0.05) correlated with important laboratory parameters as well as CV risk factors (Fig. 5A). In-depth analysis of important laboratory risk parameters unveiled a significant (*p* < 0.05) inverse correlation of plasma LDL cholesterol with CAR 16:1 and CAR 18:2 in female patients (Fig. 5B). Further, plasma HDL cholesterol significantly (*p* < 0.05) inversely correlated with CAR 10:0, CAR 14:1, CAR 16:0, CAR 18:2 in female patients, whereas LPE 0:0/18:0, LPE 0:0/18:2, LPE 0:0/20:3, LPE 18:1/0:0, LPE 18:2/0:0, LPE 20:3/0:0, LPE 22:5 and LPE 22:6 significantly (*p* < 0.05) correlated with plasma HDL cholesterol in male patients (Fig. 5C). CAR 5:0 significantly (*p* < 0.05) correlated with elevated plasma triglyceride concentrations and LPE 20:1/0:0 was inversely associated with plasma triglycerides in female patients (Fig. 5D). Of note, HbA1c levels did not correlate with platelet lipid measurements in this study and thus, did not seem to interfere with platelet lipid metabolism (Fig. 5E).

### Implementing platelet lipidomics scores for risk stratification in patients with coronary artery disease

**Figure 6:**
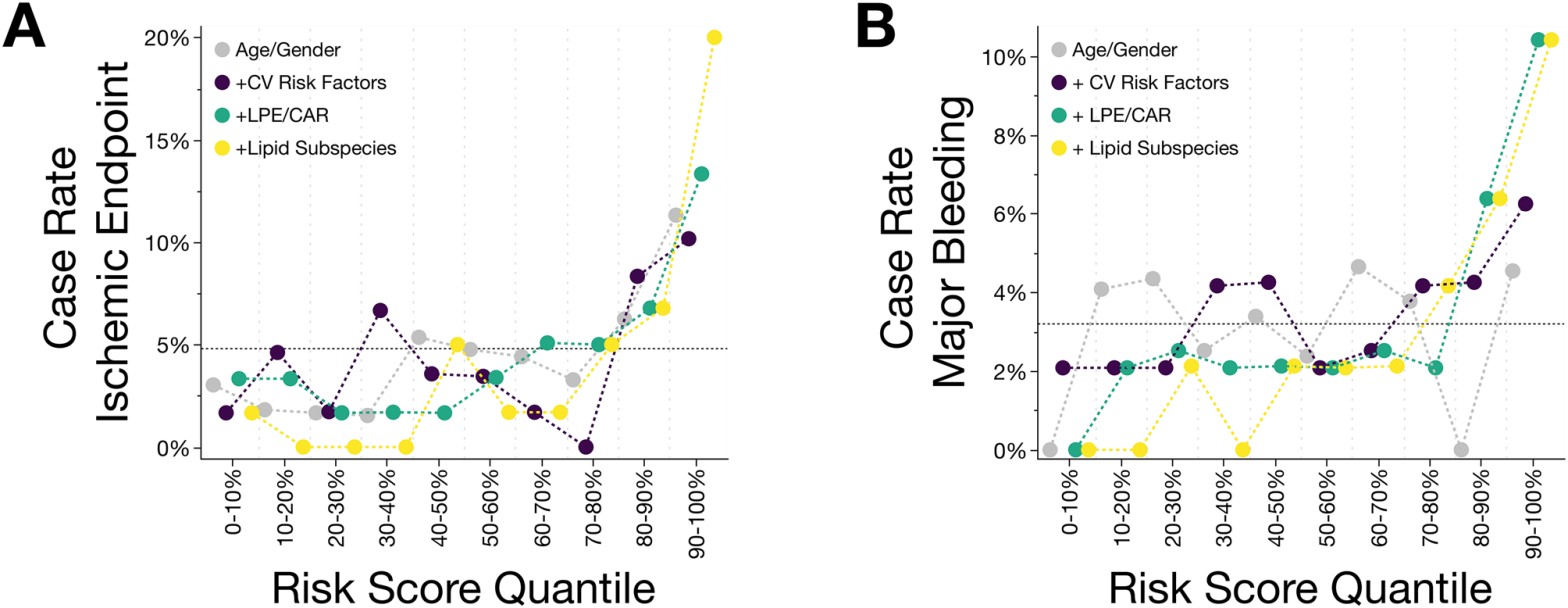
Prediction models of adverse cardiovascular events including platelet lipidomics risk scores outperformed conventional risk parameters. **(A)** Patients with CAD were partitioned into deciles according to predictive LASSO models and for each decile the fractional incidence of future CV events during the three-year follow-up is shown. The risk scores were calculated according to the included predictor variables: age/gender, CV risk factors (LVEF, LDL, HDL, triglycerides, HbA1c, platelet aggregation), platelet lipidome (mean concentrations of lysophosphatidylethanolamines (LPE) and acylcarnitines (CAR), and individual lipid LPE and CAR concentrations. The estimated mean incidence rate across the full cohort is indicated by the dotted line. (**B)** Predictive modeling of major bleeding events in patients with CAD employing different LASSO risk scores. Likewise, platelet lipids (LPE and CAR) were compared to baseline risk models (age/gender, CV risk factors) to assess the future case rates of incident bleeding.

To estimate the future CV risk to develop adverse ischemic and bleeding events, all patients enrolled into this prospective study were stratified according to the respective predictive value of individual LASSO models (Fig. 6A,B). Primarily, to assess a “baseline risk” for developing CVD or major bleeding events, a simple model containing age and gender as variables was carried out. We then analyzed for both endpoints whether quantile risk scores correlated with the case rate, implying that increasing risk score quantiles were enriched with adverse CV events in contrast to the mean incidence rates. For major bleeding events, no consistent increase in case rate with increasing risk score quantile was observed but a heterogenous spreading was depicted (Fig. 6B). Contrarily, for adverse ischemic events the 90% to 100% quantile showed a diverging case rate compared to the average rate as well as the lower risk score quantiles, respectively (Fig. 6B). In the next step, a model adding CV risk factors including LDL-cholesterol, HDL-cholesterol, triglycerides, HbA1c, LVEF and platelet function was performed for both endpoints. Risk score quantiles of the 90% to 100% subgroups were enriched with both, ischemic (Fig. 6A, case rate 10.2%) and bleeding (Fig. 6B, case rate 6.3%) events respectively, when compared to lowest risk score quantiles. Thereafter, we analyzed whether changes in the platelet lipidome might modulate the risk for future adverse CV events. Therefore, we included mean platelet LPE and CAR concentrations to the predictive models. Risk score quantiles of the lipidomics risk score showed an increasing trend with enriched incidence rates for both, ischemic and major bleeding events. For adverse CV events, the highest quantile showed a highly contrasting case rate of 13.3% compared to the average case rate (4.2%, 317% increase) as well as for the 0% to 10% quantile (3.3%, 403% increase) (Fig. 6A). Likewise, this clear contrast between highest and lowest lipidomics risk scores was observed for bleeding events, showing a 385% increase and a 495% increase for the 90% to 100% risk score quantile (case rate **10.4**%) compared to the mean case rate (2.7%) and the 0% to 10% (case rate 0%) and the 10% to 20% quantile (case rate **2.1**%), respectively (Fig. 6B). In the final predictive model, we included LPE and CAR lipid subspecies comprising 9 and 14 lipids included in LASSO analysis, respectively to estimate the risk of future adverse ischemic and bleeding events (Fig. 6A,B). In contrast to the mean case rate, incidences in the 90% to 100% risk score quantile were highest for both, adverse ischemic (case rate 20%, 476% increase) and bleeding events (case rate 10.4%, 385% increase) among all the carried-out models. Thus, platelet lipidomics risk scores outperformed the baseline model as well as conventional measurements of the CV risk. Thus, platelet lipid signatures critically increased the accuracy for CV risk prediction.

To strengthen this hypothesis, the diagnostic value of the platelet lipidomics risk score to predict adverse ischemic events was highest for mean LPE and CAR concentrations (AUC = 0.757) and LPE/CAR lipid subspecies (AUC = 0.901) when compared to conventional CV risk factors (AUC = 0.648) or age/gender (AUC = 0.579) (Table 5). Likewise, diagnostic performance to predict major bleeding events was best for the model including lipid subspecies (AUC = 0.804) and LPE/CAR concentration (AUC = 0.751) in contrast to CV risk factors (AUC = 0.633) or age/gender (AUC = 0.525) (Table 5). Subsequently, patients with a high likelihood for adverse cardiovascular events based on platelet lipidomics risk profiling, shared a high PARIS risk score (Supplementary Figure S7). Ultimately, the predictive model integrating platelet lipidic signatures unveiled a high diagnostic accuracy to distinguish between patients with adverse thrombo-ischemic (*p* = 0.001) or major bleeding events (*p* = 0.004) and those without adverse events (Supplementary Figure S8). Thus, addition of platelet lipid signatures including LPE and CAR to established CV risk factors might significantly enhance the three-year risk discrimination in patients with CAD.

**Table 5:** Enhanced diagnostic accuracy of assessing the CV risk by machine learning of the platelet lipidome.

	Ischemic Endpoint (AUC)	Bleeding (AUC)
Age/Gender	0.579	0.525
+ CV Risk Factors	0.648	0.663
+ LPE/CAR Concentration	0.757	0.751
+ Lipid Subspecies	0.901	0.804

## Discussion

The major findings of the present manuscript are: (1) Machine learning integrating CV risk factors (LDL and HDL cholesterol, triglycerides, HbA1c, LVEF, and platelet aggregation) as well as platelet lipids (LPE and CAR concentrations) could identify distinct clusters of patients with CAD. (2) Distinct platelet lipid signatures are significantly related to disease progression of CAD and addition of platelet lipids (LPE and CAR) to established CV risk factors significantly enhances the three-year risk discrimination in patients with CAD.

Our data imply that determination of the platelet lipid profile and machine learning is a valuable strategy to identify the individual risk for adverse events (thrombo-ischemic events, bleeding) in patients with CAD. Machine learning with integration of platelet lipidome data may help to tailor and to individualize antiplatelet therapy (long-term, de-escalation) in order to improve clinical outcome in CAD.

Platelet functions and platelet lipidome signatures have a significant impact on thrombo-ischemic and bleeding events in patients with CAD^6,19,21^. Current antiplatelet therapies improve clinical outcomes in patients with CAD but at the cost of an increased risk of bleeding^23,24^. The strategies for safe and effective antiplatelet therapy need to take into account the thrombotic and bleeding risk of individual patients. In the past, several score-based strategies have been suggested to provide a guide for treatment duration especially in patients receiving dual antiplatelet therapy (DAPT) to minimize both the ischemic and bleeding risk^23^. Platelet reactivity has been shown to define ischemic^6,25,26^ and bleeding^27^ events in patients undergoing coronary stenting and treatment with DAPT. However, guiding DAPT according to platelet function testing failed to improve clinical outcome after coronary stenting^28,29^.

In the present study we show that machine learning and distinct platelet lipid signatures critically increased the accuracy for CV risk prediction in patients with CAD. We identified specific clusters integrating conventional cardiovascular risk factors and platelet lipid signatures with a strong relationship to ischemic and bleeding events in the course of CAD. Integration of distinct platelet lipids (lysophosphatidylethanolamines (LPE) and acylcarnitines (CAR)) into our model led to a substantial increase of the accuracy for CV risk prediction and outperformed the baseline model as well as conventional measurements of the CV risk. We chose to integrate platelet LPE and CAR into our strategy since recently we found that both lipid subspecies were associated with adverse CV events in patients with CAD^21^. Most interestingly, the levels of both platelet lipid species improved the prediction of future case rates for ischemic and bleeding endpoints remarkably. Platelet LPE promote platelet aggregation^21,30^ and CAR have been associated with antithrombotic activity^31^. The reactivity of circulating platelets is highly dynamic and changes rapidly over time. Thus, although platelet hyperreactivity is associated with clinical prognosis in CAD, ex vivo testing of platelet function is a snapshot of platelet function which alters over time. The lipidome is remarkably stable in the context of platelet activation^12^. Less than 20% of the lipidome is altered upon activation^12^. The platelet lipidome might help to assess a sustained platelet-associated risk of patients with lower variability over time. Although it must be shown in upcoming clinical studies, the platelet lipidome might be a powerful strategy for cardiovascular risk prediction.

Thus, although we do not provide direct evidence, it is tempting to speculate that distinct platelet lipid signatures in combination with machine learning tools may be a valuable and promising strategy to assess the individual risk of patients with CAD treated with antiplatelet drugs for future thrombo-ischemic or bleeding events. A better and distinct risk profiling of patients may be a powerful tool to individualize and to define the duration of antiplatelet therapy to minimize adverse CV events and to improve clinical outcome.

### Limitations

The number of adverse events including both, thrombo-ischemic events, and major hemorrhage, was limited during the clinical follow-up period. In line with this observation, PARIS risk scores indicated a moderate cardiovascular and bleeding risk, and thus risk prediction including platelet lipid signatures might be suitable to stratify patients at modest risk. In addition, the platelet lipidome might vary with disease severity and important patient characteristics including antiplatelet or lipid lowering therapy.^19,20^ Further, severity of CAD was significantly varying among patient clusters employed for risk estimation. However, partial least squares discriminant analysis (PLS-DA) unveiled a minor impact of co-medication and severity of CAD on platelet LPE and CAR used for risk prediction in this study (Supplementary Figure S9). Nonetheless, we acknowledge that we cannot entirely preclude an impact of baseline characteristics on the observed results. Lastly, at present the underlying molecular pathophysiology of an increased cardiovascular or bleeding risk, and modulation of platelet function following changes in the platelet lipidome remain unexplored. Thus, beyond this hypothesis-generating observational study, additional research is needed to uncover the interplay of platelet lipids leading to the pathophysiology of cardiovascular diseases.

## Methods

### Study population

Five-hundred and ninety-five (595) patients with symptomatic CAD were enrolled in this consecutive, prospective study. All patients were treated for symptomatic CAD according to current international guidelines and underwent catheter-based angiography within 24 h after hospital admission. According to a standardized protocol, peripheral venipuncture was performed in patients fasting overnight for at least twelve hours. Isolation and preparation of human platelets for mass spectrometry and liquid chromatography analysis was performed as described recently^19,20^. After hospital discharge, a close-meshed clinical follow-up period over three-year was performed to screen for a composite thrombo-ischemic (i.e. cardiac death, myocardial infarction, and ischemic stroke) and bleeding events. The study was approved by the Ethics Committee at the Medical Faculty of the Eberhard Karls University and at the University Hospital of Tübingen (270/2011B01) and all patients gave written informed consent. The experiments were performed in accordance with the highest ethical standards as laid down in the Declaration of Helsinki.

### Platelet lipidomics

Untargeted lipidomics method details utilizing UHPLC-ESI-QTOF-MS/MS can be also found in the supplementary material.

Preparation, pre-processing and lipidomics analyses of platelets by mass spectrometry was performed as previously described^19,21,32^ and outlined comprehensively in the supplementary methods section. In the present study, we could verify 19 LPE and 8 CAR lipid subspecies from circulating platelets (Supplementary Table 1) and all lipids were included for further analyses of predictive risk estimation.

### Platelet function analysis

Platelet impedance aggregometry (Multiplate) was employed to analyze platelet function after stimulation of whole blood as described previously^2^ and defined in the supplementary methods section. Precisely, to define platelet hyperreactivity in patients with CAD, median data from collagen-, arachidonic acid-, adenosine diphosphate-, and thrombin-induced platelet aggregation was assessed to elucidate enhanced platelet functions independent of the external stimulant.

### Statistical analysis

Clinical data and prepared platelet lipidomics data were analyzed using JMP® Pro Version 17.1 (SAS Institute, Cary, North Carolina, USA) and different software R packages in RStudio (RStudio Inc., Boston, USA). Adjustment for age and gender was performed for all analyses and a comprehensive statistical explanation is outlined in the supplementary methods section. Mann–Whitney U test was performed for two group comparisons for non-normally distributed continuous variables, and normally distributed continuous variables were compared using student’s *t*-test, categorical parameters were compared using Chi-Square test. Mean data of individual clusters were compared using ANOVA and Tukey´s post-hoc procedure was further adopted to correct significance levels. Correlation data is based on Pearson´s product-moment correlation coefficient (*r*) and Spearman’s rank correlation coefficient (*R*). Non-normally distributed continuous data are presented as median with interquartile range (IQR), and normally distributed continuous data are represented as mean with standard deviation (SD).

To aim for a sub-phenotyping of patients with CAD and adverse events, we performed medoid clustering analyses integrating important CV risk factors including platelet lipid species. Cox regression analysis was performed to evaluate associations of platelet lipid species with adverse CV events and to test whether the platelet lipidome independently predicts incident CVD. For analysis of an increased CV or bleeding risk, we performed machine learning employing regression models including least absolute shrinkage and selection operator (LASSO). All models were trained as described in the supplementary methods section. For derivation of the predictive risk using a lipidomics risk score, we performed LASSO with tenfold cross-validation. Graphic output was performed with different software packages including RStudio and JMP.

### Supplementary Information

Supplementary Information.

## Data Availability

The data that support the findings of this study are available on reasonable request from the corresponding author.

## References

[1] Trip MD, Cats VM, van Capelle FJL, Vreeken J (1990). Platelet hyperreactivity and prognosis in survivors of myocardial infarction. N. Engl. J. Med..

[2] Droppa M (2015). Evaluation of clinical risk factors to predict high on-treatment platelet reactivity and outcome in patients with stable coronary artery disease (PREDICT-STABLE). PLoS ONE.

[3] Geisler T (2015). High platelet reactivity in patients with acute coronary syndromes undergoing percutaneous coronary intervention: randomised controlled trial comparing prasugrel and clopidogrel. PLoS ONE.

[4] Reny JL (2016). Vascular risk levels affect the predictive value of platelet reactivity for the occurrence of MACE in patients on clopidogrel systematic review and meta-analysis of individual patient data. Thromb. Haemost..

[5] Geisler T (2010). Current strategies in antiplatelet therapy–does identification of risk and adjustment of therapy contribute to more effective, personalized medicine in cardiovascular disease?. Pharmacol. Ther..

[6] Geisler T (2006). Low response to clopidogrel is associated with cardiovascular outcome after coronary stent implantation. Eur. Heart J..

[7] Geisler T (2008). The residual platelet aggregation after deployment of intracoronary stent (PREDICT) score. J. Thromb. Haemost..

[8] Gawaz M (2006). Platelets in the onset of atherosclerosis. Blood Cells Mol. Dis..

[9] Chatterjee M (2017). Regulation of oxidized platelet lipidome: Implications for coronary artery disease. Eur. Heart J..

[10] Stellos K (2012). Binding of oxidized low-density lipoprotein on circulating platelets is increased in patients with acute coronary syndromes and induces platelet adhesion to vascular wall in vivo–brief report. Arterioscler. Thromb. Vasc. Biol..

[11] Badrnya S (2014). Platelets mediate oxidized low-density lipoprotein-induced monocyte extravasation and foam cell formation. Arterioscler. Thromb. Vasc. Biol..

[12] Peng B (2018). Identification of key lipids critical for platelet activation by comprehensive analysis of the platelet lipidome. Blood.

[13] Slatter DA (2016). Mapping the human platelet lipidome reveals cytosolic phospholipase A2 as a regulator of mitochondrial bioenergetics during activation. Cell Metab..

[14] Heimerl S (2023). Quantification of bulk lipid species in human platelets and their thrombin-induced release. Sci. Rep..

[15] Goracci L (2023). A platelet lipidomics signature in patients with COVID-19. Platelets.

[16] Sun M (2022). Platelets lipidomics study of blood stasis rat model by using liquid chromatography-tandem mass spectrometry. J. Sep. Sci..

[17] Manke M-C, Ahrends R, Borst O (2022). Platelet lipid metabolism in vascular thrombo-inflammation. Pharmacol. Ther..

[18] de Jonckheere B (2023). Critical shifts in lipid metabolism promote megakaryocyte differentiation and proplatelet formation. Nat. Cardiovasc. Res..

[19] Harm T (2022). Acute coronary syndrome is associated with a substantial change in the platelet lipidome. Cardiovasc. Res..

[20] Harm T (2023). Statin treatment is associated with alterations in the platelet lipidome. Thromb. Haemost..

[21] Harm T (2023). Large-scale lipidomics profiling reveals characteristic lipid signatures associated with an increased cardiovascular risk. Clin. Res. Cardiol..

[22] Petersen-Uribe Á (2021). Platelet-derived PCSK9 is associated with LDL metabolism and modulates atherothrombotic mechanisms in coronary artery disease. Int. J. Mol. Sci..

[23] Gawaz M, Geisler T, Borst O (2023). Current concepts and novel targets for antiplatelet therapy. Nat. Rev. Cardiol..

[24] Geisler T (2023). Resumption of antiplatelet therapy after major bleeding. Thromb. Haemost..

[25] Hochholzer W (2006). Impact of the degree of peri-interventional platelet inhibition after loading with clopidogrel on early clinical outcome of elective coronary stent placement. J. Am. Coll. Cardiol..

[26] Buonamici P (2007). Impact of platelet reactivity after clopidogrel administration on drug-eluting stent thrombosis. J. Am. Coll. Cardiol..

[27] Sibbing D (2009). Platelet reactivity after clopidogrel treatment assessed with point-of-care analysis and early drug-eluting stent thrombosis. J. Am. College Cardiol..

[28] Trenk D (2012). A randomized trial of prasugrel versus clopidogrel in patients with high platelet reactivity on clopidogrel after elective percutaneous coronary intervention with implantation of drug-eluting stents. J. Am. College Cardiol..

[29] Price MJ (2011). Standard-vs high-dose clopidogrel based on platelet function testing after percutaneous coronary intervention: the GRAVITAS randomized trial. Jama.

[30] Xiao H, Siddiqui RA, Al-Hassani MH, Sliva D, Kovacs RJ (2001). Phospholipids released from activated platelets improve platelet aggregation and endothelial cell migration. Platelets.

[31] Deguchi H (2015). Acylcarnitines are anticoagulants that inhibit factor Xa and are reduced in venous thrombosis, based on metabolomics data. Blood.

[32] Cebo M (2021). Untargeted UHPLC-ESI-QTOF-MS/MS analysis with targeted feature extraction at precursor and fragment level for profiling of the platelet lipidome with ex vivo thrombin-activation. J. Pharm. Biomed. Anal..

