# Supplementary material for: Machine learning insights into thrombo-ischemic risks and bleeding events through platelet lysophospholipids and acylcarnitine species
Source: Sci Rep. 2024 Mar 13;14:6089. doi: 10.1038/s41598-024-56304-x (PMC10937715; doi:10.1038/s41598-024-56304-x)
Supplement: Supplementary file 1 — Supplementary Information. [file 41598_2024_56304_MOESM1_ESM.docx]

**Machine Learning Insights into Thrombo-ischemic Risks and Bleeding Events through Platelet Lysophospholipids and Acylcarnitine Species**

**Short title:** Machine learning and platelet lipidome

Tobias Harm^1^, Xiaoqing Fu^2^, Moritz Frey^1^, Kristina Dittrich^2^, Adrian Brun^2^, Tatsiana Castor^1^, Oliver Borst^1^, Karin Anne Lydia Müller^1^, Tobias Geisler^1^, Dominik Rath^1^, Michael Lämmerhofer^2^, Meinrad Paul Gawaz^1*^

^1^Department of Cardiology and Angiology, University Hospital Tübingen, Eberhard Karls University Tübingen, Otfried-Müller-Straße 10, 72076 Tübingen, Germany ^2^Institute of Pharmaceutical Sciences, Eberhard Karls University Tübingen, Auf der Morgenstelle 8, 72076 Tübingen, Germany

***Correspondence to:**

**Meinrad Paul Gawaz, MD**

Department of Cardiology and Angiology

University Hospital Tübingen, Eberhard Karls University Tübingen

Otfried-Müller-Str. 10

72076 Tübingen, Germany

Tel.: +49 (0) 7071 29 83688

Fax: +49 (0) 7071 29 5749

E-Mail: [meinrad.gawaz@med.uni-tuebingen.de](mailto:meinrad.gawaz@med.uni-tuebingen.de)

**Supplementary Material and Methods**

**Study population**

This consecutive and prospective study was enrolled at University Hospital of Tübingen and a median follow-up period over three years was chosen to screen for thrombo-ischemic and bleeding events. All patients were treated for symptomatic coronary artery disease (CAD) according to international guidelines. The severity of CAD was analyzed by catheter angiography within 24h after hospital admission and patients without relevant CAD (hemodynamic relevant ≥50% lumen narrowing stenosis) were excluded from this study.

**Cardiovascular risk stratification**

A standardized questionnaire on medication history, dietary items, current and past smoking status was implemented for all patients enrolled into this consecutive, prospective study. Cardiovascular (CV) risk factors were assessed according to standard procedures as described previously by international guidelines. All CV risk factors and disease history were obligatorily confirmed by reviewing the individual medical records. During the three-year follow-up, the incidence of myocardial infarction or ischemic stroke was classified according to current European guidelines.

Cardiac death summarized incidences of sudden cardiac death (SCD) and aborted SCD, as aborted SCD was defined by resuscitation after cardiac arrest or appropriate defibrillator discharge. Likewise, bleeding events were stratified according to the International Society on Thrombosis and Haemostasis (ISTH) and major bleeding events were defined as follows: a fall in hemoglobin (>2g/dl) or leading to transfusion of whole blood or red cells, critical bleeding (e.g. pericardial, intracranial) or fatal bleeding.

**Analyses of platelet lipid signatures by an untargeted UHPLC-MS/MS approach**

Platelet-rich plasma (PRP) was obtained from citrated whole blood by centrifugation at 430xg for 20 min, further diluted and buffered with Tyrodes-HEPES (HEPES-2.5mM; NaCl-150mM; KCl-1mM; NaHCO_3_-2.5mM; NaH_2_PO_4_-0.36mM; Glucose- 5.5mM; BSA-1mg/ml; pH 6.5) as recently described^1,2^. PRP/Tyrodes cell suspension was again centrifuged at 900xg for 10 min to isolate platelets. Platelet isolates (2 x 10^8^) were then resuspended in Tyrodes-HEPES solvent (pH 7.4; supplemented with CaCl_2_-1mM; MgCl_2_-1mM) for a further washing step and platelet pellets isolated for lipidomics analysis. For lipid extraction and analysis, platelet samples were divided into 22 processing batches. Prior addition of the extraction solvent, stable isotope labelled internal standards were added to the platelet pellets and allowed to incubate. The following internal standards were employed: EquiSPLASH® LIPIDOMIX® (0.5%), AA(d11) (100 ng/mL), palmitoyl-L-carnitine(d3) (500 ng/mL), 15:0-18:1(d7) PA (500 ng/mL), Cholesterol(d7) (2000 ng/mL), LysoSM d18:1(d9) (500 ng/mL), sphingosine-1-phosphate d18:1(d7) (5000 ng/mL), and 18:1(d7) Chol Ester (10000 ng/mL: 500 ng/mL from LIPIDOMIX® and additional spike of 9500 ng/mL). Lipid extraction was then performed by a monophasic extraction protocol (with 2-propanol/water 90:10, v/v) as reported previously^3^. Lipid extracts were evaporated and reconstituted, and then analyzed employing a reversed-phase liquid chromatography mass spectrometry (RP-UHPLC-ESI-QTOF-MS/MS) approach^3^,^4^. In both, positive and negative ion mode, acquisition was performed using data independent sequential window acquisition of all theoretical fragment ion spectra (SWATH) **(Supplementary Table S1 in ref.**^1^**)**. Matrix-matched quality control (QC) samples were implemented to all batches to enable post-acquisition data normalization. After quality verification of the analytical runs, sequential batches were processed with MS-DIAL software allowing peak picking, background subtraction, batch alignment, and lipid annotation through MS/MS spectra deconvolution. Results were then matched with the LipidBlast spectral database^4^. Thereafter, reference batches were aligned by an in-house developed inter-batch feature alignment tool (VBA), for both positive and negative mode, respectively. Annotation of lipid subspecies were revised and corrected manually by inspecting all features under consideration of the retention time, class specific fragments, homologous retention time series and MS-DIAL scores. The final reference feature lists were used for targeted data extraction of all batches using Sciex MultiQuant (Concord, Ontario, Canada). Sum extraction of all adduct types and charge states ([M-2], [M-1], [M], [M+1], [M+2]) was performed for all standards and peak heights of all features were merged into one data matrix. In the matrix, missing values imputation was performed using a random forest algorithm in R (package *missForest)*^5^. For multiple adducts of the same lipid species peak heights were combined and final data were adjusted for carbon isotopes by type I isotopic correction^6^. Data were normalized based on QC samples using a random forest (SERRF) algorithm^7^ and lipids with a CV > 30% in QC samples were removed. Finally, lipid concentrations in pmol/10^6^ platelets were calculated by one-point calibration with class-specific internal standards for relative quantification. For this study, only the results for LPEs and acylcarnitines were considered. For quantification of LPEs 1-(9Z -octadecenoyl)(d7)-2-hydroxy-sn-glycero-3-phosphoethanolamine (18:1(d7) LPE) and for acylcarnitines palmitoyl-L-carnitine(d3) were employed as internal standards. The method performance of this validated untargeted analytical LC-MS/MS lipidomics method is described in detail by a previous study^8^.

**Statistical analysis**

To perform a pathophysiology-based sub-phenotyping of patients with CAD, we performed medoid clustering analyses using R-package ‘cluster’. Therefore, patients baseline characteristics data were standardized, and Gower distances were computed. Analysis of dendrogram and silhouette-widths was performed to find the optimal cluster aggregate **(Supplementary Figure S1).** Thereafter, clustering was performed with partitions around medoids using the R-package ‘cluster’ package algorithm, which is a robust procedure in the setting of population data^9,10^. In the graphic output of radar chars, negative z-scores were directionally flipped (-1*z-score) to depict radar chards with polygon areas related to adverse variable effects. A comprehensive comparison of cluster characteristics is summarized in **Supplementary Table 2** and **Supplementary Figures S5&S6.** CV endpoints including major bleeding and thrombo-ischemic events were analyzed in the follow-up data of individual clusters using survival analysis and Cox proportional hazard models. A comparison of the cumulative risks for reaching the individual endpoints was performed with Wilcoxon tests. Therefore, hazards ratios and confidence intervals were calculated by analyzing the significance level of the correlation coefficient between Kaplan-Meier-transformed survival time and scaled Schoenfeld residuals. For analysis of an increased cardiovascular or bleeding risk, we performed machine learning employing least absolute shrinkage and selection operator (LASSO), ridge, elastic net, forward selection, fit stepwise, neural boosted, forward selection, decision tree and naïve bayes models. Input data were randomly split into test data (20%, n=119) and training data (80%, n=476). Individual models were trained using a 5× repeated 10-fold cross-validation loop and the final model was fit on all training data emitting a mean absolute error (MAE) of each individual model. LASSO was implemented for further analyses because of its good interpretability and a superior MAE. L_1_-regularised Cox regression (LASSO) algorithm was implemented for CV risk assessment and optimal λ was chosen by 10-fold cross-validation. Likewise, adjustment for age and gender was implemented. We then applied the trained models to the individuals from the overall cohort. The final risk score of adverse CV events for each individual was derived from LASSO prediction (likelihood score) and patients were split into equally sized quantiles, accordingly. For partial least squares discriminant analyses (PLS-DA) all lipids were included into the multivariate analysis. To assess the impact of co-medication on the platelet lipidome, orthogonal partial least squares discriminant analyses (OPLS-DA) was employed and for multiple group comparison of CAD type a PLS-DA model was performed.

**Lipid selection and prediction of the cardiovascular risk**

Where indicated, we integrateted mean concentrations of LPE and CAR lipids respectively, that were recently shown to be upregulated in patients with adverse events^11^. For regression analyses and prediction models, we included all individual LPE/CAR subspecies as specified. L_1_-regularized Cox regression with a least absolute shrinkage and selection operator (LASSO) algorithm was implemented for selection of platelet lipid signatures in the process of assessing new risk factors^12^. The penalized regression intends to shrink coefficients to prevent overfitting in the setting of large-scale omics data. To carry out different predictive models, ideal hyperparameter λ was chosen by a 5x repeated 10-fold cross-validation. Therefore, CV risk factors including platelet lipidomics data were divided into ten even partitions and all variables were fitted using nine of these partitions to determine the mean partial likelihood. CV risk factors sharing a nonzero coefficient from different random data partitions with optimal λ were included for further analysis of the predictive risk. To estimate the CV risk in patients with CAD, we implemented different models using distinct platelet lipids (LPE/CAR) in addition to conventional CV risk factors.

Thus, lipid subspecies included 19 LPE and 8 CAR and those lipids were integrated into LASSO analysis. Further, confidence intervals of the final lipidomics model were generated utilizing percentile bootstrap (100 repetitions) analysis. Prediction curves were assessed by adding platelet lipidomics data to the baseline models (age and gender, conventional CV risk factors, respectively).

**Platelet impedance aggregometry**

Platelets of hirudinized whole blood (300 µl) in patients with coronary artery disease were stimulated with 20 µL adenosine diphosphate (ADP) 6.5 µM, arachidonic acid (AA) 484 µM, thrombin receptor activating peptide-6 (TRAP) 32µM, or collagen 3.2 µg/mL. Thereafter, platelet impedance aggregometry was performed using Multiplate Analyzer (F. Hoffmann-La Roche Ltd., Basel, Schweiz). Assessment of platelet impedance was performed over 6 minutes and means of area under the curve (AUC) of the tested aliquots were integrated into further prediction analyses. A detailed comparison of platelet impedance aggregometry between clusters of CAD patients is highlighted in **Supplementary Figure S6.**

**Supplementary Table S1.** Reporting of identifications by MS of platelet lipid species included in CV prediction models

| **lipid species**  **(w/ side chain)** | **Class** | **formula** | **ion mode** | **t_R,reference_**  **(min)** | **precursor**  **ion type** | **precursor**  **exact *m/z*** | **precursor**  **accurate** | **mass accuracy**  **(ppm)** | **MS/MS fragments** |
| --- | --- | --- | --- | --- | --- | --- | --- | --- | --- |
| **CAR 5:0** | ACar | C12H23NO4 | pos | 0.48 | [M+H]+ | 246.1700 | 246.1705 | 2.03 | 85.0284 |
| **CAR 8:0** | ACar | C15H29NO4 | pos | 0.54 | [M+H]+ | 288.2170 | 288.2177 | 2.39 | 85.0284 |
| **CAR 10:0** | ACar | C17H33NO4 | pos | 0.62 | [M+H]+ | 316.2483 | 316.2477 | 1.77 | 85.0284 |
| **CAR 12:0** | ACar | C19H37NO4 | pos | 0.78 | [M+H]+ | 344.2796 | 344.2778 | 5.25 | 85.0284 |
| **CAR 14:0** | ACar | C21H41NO4 | pos | 1.01 | [M+H]+ | 372.3109 | 372.3114 | 1.40 | 85.0284 |
| **CAR 14:1** | ACar | C21H39NO4 | pos | 0.90 | [M+H]+ | 370.2952 | 370.2948 | 0.99 | 85.0284 |
| **CAR 16:1** | ACar | C23H43NO4 | pos | 1.15 | [M+H]+ | 398.3265 | 398.3252 | 3.22 | 85.0284 |
| **CAR 18:2** | ACar | C25H45NO4 | pos | 1.30 | [M+H]+ | 424.3422 | 424.3394 | 6.52 | 85.0284 |
| **LPE 16:0/0:0** | LPE | C21H44NO7P | neg* | 1.50 | [M-H]- | 452.2783 | 452.2782 | 0.14 | 255.2330; 214.0486; 196.0380 |
| **LPE 0:0/18:0** | LPE | C23H48NO7P | neg* | 2.13 | [M-H]- | 480.3096 | 480.3105 | 1.87 | 283.2643; 214.0486; 196.0380 |
| **LPE 18:0/0:0** | LPE | C23H48NO7P | neg* | 2.31 | [M-H]- | 480.3096 | 480.3090 | 1.31 | 283.2643; 214.0486; 196.0380 |
| **LPE 0:0/18:1** | LPE | C23H46NO7P | neg* | 1.52 | [M-H]- | 478.2940 | 478.2915 | 5.27 | 281.2486; 214.0486; 196.0380 |
| **LPE 18:1/0:0** | LPE | C23H46NO7P | neg* | 1.66 | [M-H]- | 478.2940 | 478.2918 | 4.52 | 281.2486; 214.0486; 196.0380 |
| **LPE 0:0/18:2** | LPE | C23H44NO7P | neg* | 1.20 | [M-H]- | 476.2783 | 476.2752 | 6.49 | 279.2330; 214.0486; 196.0380 |
| **LPE 18:2/0:0** | LPE | C23H44NO7P | neg* | 1.23 | [M-H]- | 476.2783 | 476.2778 | 1.13 | 279.2330; 214.0486; 196.0380 |
| **LPE 20:0/0:0** | LPE | C25H52NO7P | neg* | 3.02 | [M-H]- | 508.3409 | 508.3390 | 3.83 | 311.2956; 214.0486; 196.0380 |
| **LPE 20:1/0:0** | LPE | C25H50NO7P | neg* | 2.44 | [M-H]- | 506.3253 | 506.3278 | 4.98 | 309.2799; 214.0486; 196.0380 |
| **LPE 0:0/20:3** | LPE | C25H46NO7P | neg* | 1.32 | [M-H]- | 502.2940 | 502.2933 | 1.33 | 305.2486; 214.0486; 196.0380 |
| **LPE 20:3/0:0** | LPE | C25H46NO7P | neg* | 1.44 | [M-H]- | 502.2940 | 502.2942 | 0.39 | 305.2486; 214.0486; 196.0380 |
| **LPE 0:0/20:4** | LPE | C25H44NO7P | neg* | 1.13 | [M-H]- | 500.2783 | 500.2776 | 1.39 | 303.2330; 214.0486; 196.0380 |
| **LPE 0:0/20:5** | LPE | C25H42NO7P | neg* | 0.99 | [M-H]- | 498.2627 | 498.2615 | 2.47 | 301.2173; 214.0486; 196.0380 |
| **LPE 0:0/22:4** | LPE | C27H48NO7P | neg* | 1.58 | [M-H]- | 528.3096 | 528.3092 | 0.76 | 331.2643; 214.0486; 196.0380 |
| **LPE 22:4/0:0** | LPE | C27H48NO7P | neg* | 1.67 | [M-H]- | 528.3096 | 528.3084 | 2.29 | 331.2643; 214.0486; 196.0380 |
| **LPE 0:0/22:5** | LPE | C27H46NO7P | neg* | 1.27 | [M-H]- | 526.2940 | 526.2930 | 1.84 | 329.2486; 214.0486; 196.0380 |
| **LPE 0:0/22:6** | LPE | C27H44NO7P | neg* | 1.13 | [M-H]- | 524.2783 | 524.2782 | 0.18 | 327.2330; 214.0486; 196.0380 |
| **LPE P-16:0** | LPE | C21H44NO6P | pos* | 1.75 | [M+H]+ | 436.2834 | 436.2824 | 2.38 | 420.2873; 284.2948; 266.2842 |
| **LPE P-18:0** | LPE | C23H48NO6P | pos* | 2.62 | [M+H]+ | 464.3147 | 464.3121 | 5.55 | 448.3186; 312.3261; 294.3155 |

|  | **2-1** | **3-1** | **4-1** | **5-1** | **6-1** | **3-2** | **4-2** | **5-2** | **6-2** | **4-3** | **5-3** | **6-3** | **5-4** | **6-4** | **6-5** |
| --- | --- | --- | --- | --- | --- | --- | --- | --- | --- | --- | --- | --- | --- | --- | --- |
| Female, n | 0.075 | 0.051 | 0.317 | 0.236 | 0.558 | 0.987 | **0.135** | 0.928 | 0.104 | 0.506 | 0.935 | 0.095 | 0.658 | 0.245 | 0.157 |
| Age, years | 0.336 | 0.410 | **0.044** | 0.256 | 0.456 | 0.999 | **1.3*10*^-4^** | 0.996 | 0.986 | **1.1*10*^-4^** | 0.971 | 0.959 | **3.3*10^-4^** | **0.009** | 0.999 |
| Body mass index | 0.926 | 0.577 | **2*10^-7^** | 0.561 | 0.998 | 0.996 | **1.1*10*^-4^** | 0.967 | 0.923 | **1.5*10*^-4^** | 0.998 | 0.782 | **0.024** | **0.002** | 0.688 |
| **Cardiovascular risk factors** | | | | | | | | | | | | | | | |
| Arterial hypertension, n | 0.555 | 0.094 | **0.040** | 0.752 | **0.824** | **0.341** | 0.134 | 0.946 | 0.914 | 0.449 | 0.388 | 0.491 | 0.163 | 0.243 | 0.957 |
| Hyperlipidemia, n | 0.108 | **0.015** | 0.852 | **0.023** | 0.762 | 0.520 | 0.228 | 0.466 | 0.272 | 0.064 | 0.809 | 0.152 | 0.081 | 0.694 | 0.141 |
| Diabetes mellitus, n | **1.1*10^-6^** | **0.005** | **1.8*10^-12^** | **8.1*10^-7^** | 0.749 | **0.022** | **0.018** | 0.335 | **0.027** | **2.9*10^-6^** | **0.004** | 0.271 | 0.283 | **4*10^-4^** | **0.008** |
| Current smoking, n | 0.807 | 0.263 | 0.107 | 0.113 | 0.936 | 0.433 | 0.088 | 0.149 | 0.829 | **0.009** | 0.353 | 0.490 | **0.007** | 0.414 | 0.205 |
| Ex Smoking > 6 mo, n | 0.177 | 0.511 | 0.916 | 0.356 | 0.342 | **0.049** | 0.286 | 0.963 | 0.114 | 0.501 | 0.116 | 0.518 | 0.386 | 0.331 | 0.141 |
| Obesity, n | 0.721 | 0.132 | **1.8*10^-9^** | 0.311 | 0.355 | 0.310 | **2.2*10^-7^** | 0.404 | 0.282 | **2.1*10^-6^** | 0.967 | 0.117 | 4*10^-4^ | **1.4*10^-4^** | 0.135 |
| Atrial Fibrillation, n | **0.018** | 0.725 | 0.341 | **2.4*10^-4^** | 0.086 | **0.041** | **0.004** | 0.067 | 0.795 | 0.214 | **2.2*10^-4^** | 0.132 | 2.2*10^-5^ | **0.024** | 0.336 |
| Previous CABG, n | **0.008** | 0.104 | **0.016** | **0.027** | 0.127 | 0.219 | 0.839 | 0.767 | 0.687 | 0.348 | 0.489 | 0.798 | 0.907 | 0.779 | 0.848 |
| Previous MI, n | **9*10^-4^** | **0.001** | **4.4*10^-5^** | **0.022** | 0.511 | **0.001** | 0.088 | **0.013** | **0.011** | 0.245 | 0.895 | 0.306 | 0.312 | 0.116 | 0.383 |
| **Laboratory parameters** | | | | | | | | | | | | | | | |
| LDL-cholesterol (mg/dL) | **9.8*10^-11^** | **9.8*10^-11^** | **9.8*10^-11^** | **9.8*10^-11^** | **9.8*10^-11^** | **2.4*10^-5^** | **4.8*10^-4^** | **0.010** | 0.796 | **9.8*10^-11^** | 0.999 | **8*10^-4^** | **6*10^-10^** | 0.766 | **8.3*10^-4^** |
| HDL-cholesterol (mg/dL) | **4.8*10^-5^** | **1.3*10^-4^** | **9.8*10^-11^** | **3.1*10^-6^** | 0.999 | 0.989 | **1.8*10^-4^** | 0.735 | 0.101 | **2.3*10^-6^** | 0.362 | 0.191 | 0.165 | **3*10^-6^** | **0.012** |
| Triglycerides (mg/dL) | 0.884 | 0.252 | **9.8*10^-11^** | 0.999 | 0.999 | 0.950 | **9.8*10^-11^** | 0.970 | 0.999 | **9.8*10^-11^** | 0.635 | 0.951 | **9.8*10^-11^** | **9.8*10^-11^** | 0.999 |
| Total cholesterol (mg/mL) | **9.8*10^-11^** | **9.8*10^-11^** | **2.5*10^-10^** | **9.8*10^-11^** | **2*10^-6^** | **0.004** | **5.6*10^-7^** | **0.023** | 0.149 | **9.8*10^-11^** | 0.999 | **8.9*10^-5^** | 9.9*10^-11^ | 0.891 | **2.5*10^-4^** |
| HbA1c (%) | **2.5*10^-5^** | 0.523 | **9.8*10^-11^** | **0.001** | 0.998 | **0.014** | **0.011** | 0.999 | 0.208 | **9.9*10^-10^** | 0.071 | 0.997 | 0.067 | **3*10^-4^** | 0.262 |
| Renal function (GFR) | **0.036** | 0.959 | 0.999 | 0.995 | 0.999 | 0.230 | 0.154 | 0.477 | 0.565 | 0.996 | 0.999 | 0.997 | 0.999 | 0.999 | 0.999 |
| **LVEF (%)** | **9.8*10^-11^** | 0.380 | 0.999 | 0.999 | **0.005** | **9.8*10^-11^** | **9.8*10^-11^** | **9.8*10^-11^** | **9.8*10^-11^** | 0.805 | 0.519 | **9*10^-5^** | 0.993 | **0.004** | **0.031** |
| **Platelets (10^9^/l)** | 0.992 | **0.015** | 0.999 | 0.956 | 0.982 | 0.161 | 0.996 | 0.999 | 0.915 | 0.062 | 0.634 | 0.163 | 0.971 | 0.985 | 0.848 |
| Platelet Aggregation (AUC) | 0.920 | **0.041** | 0.994 | **9.8*10^-11^** | 0.091 | 0.549 | 0.736 | **9.8*10^-11^** | 0.332 | **0.029** | **9.8*10^-11^** | 0.870 | **9.8*10^-11^** | 0.054 | **9.8*10^-11^** |
| **Platelet lipidomics** | | | | | | | | | | | | | | | |
| LPE (pmol/10^9^ platelets) | 0.999 | 0.999 | 0.993 | 0.998 | **9.8*10^-11^** | 0.999 | 0.999 | 0.999 | **9.8*10^-11^** | 0.999 | 0.999 | **9.8*10^-11^** | 0.999 | **9.8*10^-11^** | **9.8*10^-11^** |
| CAR (pmol/10^9^ platelets) | **2.2*10^-5^** | 0.256 | 0.968 | **9.8*10^-11^** | **8.7*10^-4^** | **3.7*10^-10^** | **0.007** | **0.001** | 1.3*10^-9^ | 0.088 | **9.8*10^-11^** | **0.042** | **4.2*10^-10^** | **2.8*10^-4^** | **9.8*10^-11^** |

**Supplementary Table S2.** Post-hoc tests for comparison of cluster means with numbers indicating p-values for all pairwise comparisons computed with Tukey’s test. Signficant comparisons (p<0.05) are highlighted

**Supplementary Table S3.** Cox proportional hazard model estimating the thrombo-ischemic risk including platelet lipid signatures. Variables significantly (p<0.05) contributing to the prediction of thrombo-ischemic events during the three-year follow-up are highlighted. LPE, Lysophosphatidylethanolamine; CAR, acylcarnitine; CI, confidence interval

| **Model 1: Ischemic Endpoint; p=0.031** | **Hazard Ratio (95% CI)** | **p-Value** |
| --- | --- | --- |
| LPE | 0.39 (0.75-90.88) | 0.479 |
| CAR | 23.40 (1.79-306.75) | **0.016** |
| LPE/CAR Ratio | 97.35 (2.84-5000) | **0.015** |

**Supplementary Table S4.** Cox proportional hazard mode assessing the bleeding risk including platelet lipid signatures. Variables significantly (p<0.05) contributing to the prediction of major bleeding events during the three-year follow-up are highlighted. LPE, Lysophosphatidylethanolamine; CAR, acylcarnitine; CI, confidence interval

| **Model 2: Major Bleeding; p=0.049** | **Hazard Ratio (95% CI)** | **p-Value** |
| --- | --- | --- |
| LPE | 2.51 (0.16-18.1) | 0.448 |
| CAR | 127.56 (5.66-2386.41) | **0.002** |
| LPE/CAR Ratio | 5.9 (0.17-267.59) | 0.617 |

**Supplementary Figure S1. (A)** Plot of within-groups sum of squares against number of clusters to identify the optimal count of clusters to sub-phenotype patients with CAD. Thus, the results of the sum of squares for 1-10 groups using medoid clustering reflect that after 6 clusters the observed difference in the within-cluster dissimilarity is not significant. Therefore, the optimal number of six clusters is highlighted. (**B)** The expected k-medoids plot of the cluster analysis highlighting the distribution of six clusters characterized by CV risk factors.

**Supplementary Figure S2.** Manhattan plot displays abundance of individual LPE and CAR species integrated for risk assessment using platelet lipidomics data. Dots represent median concentrations of lipid species detected in all patients within this study. Cluster-specific abundance of platelet lipids based on medoids from stratification of CAD patients. The most abundant lipid detected among patient cluster 1 to 5 was LPE 18:0/0:0, whereas LPE 0:0/20:4 exhibited highest concentrations within patient cluster 6. Lipids were colored based on median overall concentrations and characteristic lipids with highest abundance within each cluster were highlighted.

**Supplementary Figure S3.** Kaplan–Meier curves showing cluster-specific probability to develop adverse ischemic and major bleeding events (**A**; composite EP) and the increased risk of all-cause mortality during the three-year follow-up (**B**).

**Supplementary Figure S4.** Kaplan–Meier curves plotting the three-year probability of (**A)** ischemic events, and (**B)** major bleeding according to disease severity of coronary artery disease (CAD). There was no significant difference in both, the cardiovascular and bleeding risk between patients with ST-elevation myocardial infarction (STEMI), non-STE-elevation acute coronary syndrome (NSTE-ACS), or chronic coronary syndrome (CCS).

**Supplementary Figure S5.** Comparison of mean CV risk factors including (**A**) LDL-cholesterol, (**B**) HDL-cholesterol, (**C**) plasma triglycerides, (**D**) total plasma cholesterol, (**E**) HbA1c, and (**F**) LV ejection fraction. Boxes denote the 25th and 75th percentiles as well as the median alongside whiskers (1.5xIQR) and the highest and lowest outliers. Differences between clusters were tested with one-way ANOVA and showed statistical significance (p<0.05).

**Supplementary Figure S6.** Comparison of individual platelet aggregation assays among clusters of patients with CAD. (**A**) collagen-induced (n=254), (**B**) arachidonic acid-induced (n=595), (**C**) adenosine diphosphate-induced (n=595), and (**D**) thrombin-induced (n=595) platelet aggregation was significantly (p<0.05) different among individual clusters. Boxes denote the 25th and 75th percentiles as well as the median, whiskers show 1.5xIQR and data points depict highest and lowest individual outliers. Differences between clusters were tested with one-way ANOVA and showed statistical significance (p<0.05)

**Supplementary Figure S7. Estimation of both, thrombo-ischemic events and major bleeding integrating platelet lipid signatures shows a good agreement with reliable risk scores.** All patients were grouped according to percentiles of the lipidomics risk model including LPE and CAR subspecies to estimate (**A)** ischemic events and (**B)** major hemorrhage and were plotted against PARIS ischemic or bleeding risk score. Patients with an increased cardiovascular risk based on assessment of the platelet lipidome- exhibited a high PARIS score indicating a significant agreement between risk scores.

**
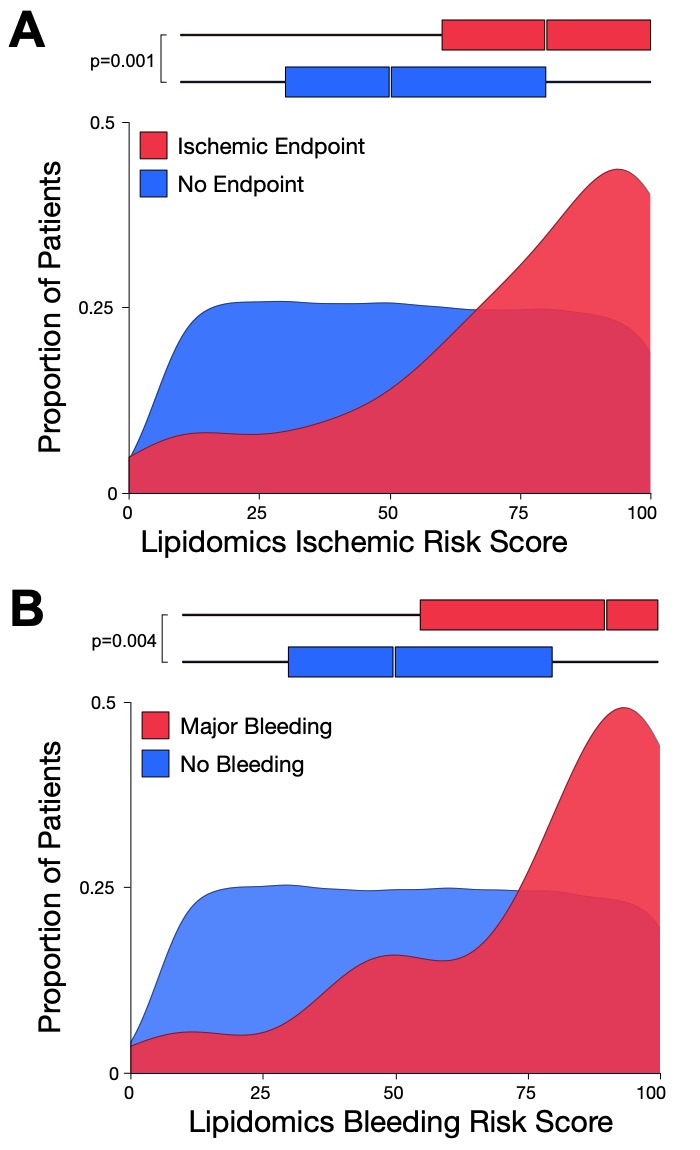
**

**Supplementary Figure S8.** Patients were grouped based on tansformed likelihood ratio from LASSO model including platelet LPE and CAR subspecies (x-axis). The individual models showed a high accuracy to distinguish between patients with adverse events (red) or those without (blue) adverse events during the clinical follow-up. The proportion of patients (y-axis) with (**A**) ischemic, or (**B**) major bleeding events was critically increased in those patients with a high risk according to platelet lipidomics score. Thus, the models significantly (p<0.05) distinguished between patients with and without adverse events during the three-year follow-up.


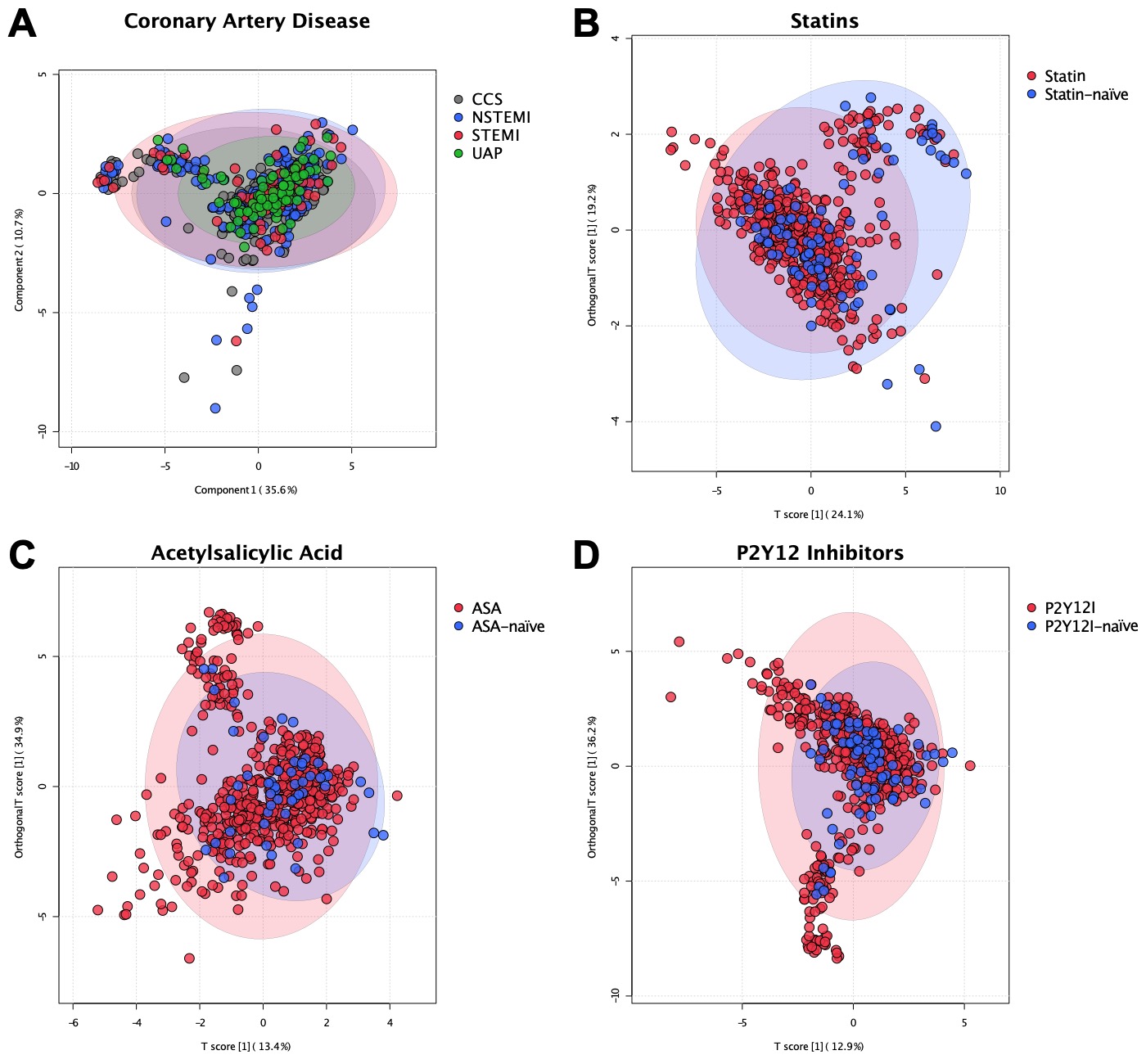


**Supplementary Figure** **S9.** **Effect of disease severity and co-medication (statins, acetylsalicylic acid (ASA), and P2Y12 inhibitors) on the platelet lipidome. (A-D)** Supervised partial least squares discriminant analyses (PLS-DA) assessing the impact of statin, ASA and P2Y12-inhibitor treatment, and type of CAD on LPE and CAR species. Data were based on normalized peak intensities of LPE and CAR in all patients enrolled into this study (n=595). The subcohorts are colored and labelled, accordingly. A homogenous spreading of the patient subgroups implements a minor influence of assessed pharmaceutical treatment and disease severity on the platelet lipids incorporated for risk estimation in this study.

**References**

1 Harm, T. *et al.* Acute coronary syndrome is associated with a substantial change in the platelet lipidome. *Cardiovasc Res* 118, 1904-1916 (2022). <https://doi.org/10.1093/cvr/cvab238>

2 Harm, T. *et al.* Statin Treatment Is Associated with Alterations in the Platelet Lipidome. *Thromb Haemost* (2023). <https://doi.org/10.1055/s-0043-1764353>

3 Calderón, C., Sanwald, C., Schlotterbeck, J., Drotleff, B. & Lämmerhofer, M. Comparison of simple monophasic versus classical biphasic extraction protocols for comprehensive UHPLC-MS/MS lipidomic analysis of Hela cells. *Anal Chim Acta* 1048, 66-74 (2019). <https://doi.org/10.1016/j.aca.2018.10.035>

4 Tsugawa, H. *et al.* MS-DIAL: data-independent MS/MS deconvolution for comprehensive metabolome analysis. *Nat Methods* 12, 523-526 (2015). <https://doi.org/10.1038/nmeth.3393>

5 Stekhoven, D. J. & Bühlmann, P. MissForest—non-parametric missing value imputation for mixed-type data. *Bioinformatics* 28, 112-118 (2011). <https://doi.org/10.1093/bioinformatics/btr597>

6 Wang, M., Wang, C. & Han, X. Selection of internal standards for accurate quantification of complex lipid species in biological extracts by electrospray ionization mass spectrometry—What, how and why? *Mass Spectrometry Reviews* 36, 693-714 (2017). <https://doi.org/https://doi.org/10.1002/mas.21492>

7 Fan, S. *et al.* Systematic Error Removal Using Random Forest for Normalizing Large-Scale Untargeted Lipidomics Data. *Analytical Chemistry* 91, 3590-3596 (2019). <https://doi.org/10.1021/acs.analchem.8b05592>

8 Drotleff, B., Illison, J., Schlotterbeck, J., Lukowski, R. & Lämmerhofer, M. Comprehensive lipidomics of mouse plasma using class-specific surrogate calibrants and SWATH acquisition for large-scale lipid quantification in untargeted analysis. *Analytica Chimica Acta* 1086, 90-102 (2019). <https://doi.org/https://doi.org/10.1016/j.aca.2019.08.030>

9 Wagner, R. *et al.* Pathophysiology-based subphenotyping of individuals at elevated risk for type 2 diabetes. *Nature Medicine* 27, 49-57 (2021). <https://doi.org/10.1038/s41591-020-1116-9>

10 Reynolds, A. P., Richards, G., de la Iglesia, B. & Rayward-Smith, V. J. Clustering Rules: A Comparison of Partitioning and Hierarchical Clustering Algorithms. *Journal of Mathematical Modelling and Algorithms* 5, 475-504 (2006). <https://doi.org/10.1007/s10852-005-9022-1>

11 Harm, T. *et al.* Large-scale lipidomics profiling reveals characteristic lipid signatures associated with an increased cardiovascular risk. *Clin Res Cardiol* (2023). <https://doi.org/10.1007/s00392-023-02260-x>

12 Goeman, J. J. L1 penalized estimation in the Cox proportional hazards model. *Biom J* 52, 70-84 (2010). <https://doi.org/10.1002/bimj.200900028>
